# PI3K/Akt/mTOR pathway expression profiling reveals age- and subtype-specific molecular heterogeneity in the Nigerian breast cancer landscape

**DOI:** 10.3389/fonc.2026.1766066

**Published:** 2026-03-11

**Authors:** Magdalene Eno Udobi, Shalom Nwodo Chinedu, Israel Sunmola Afolabi, Kevin Nwabueze Ezike, Cynthia Nwamaka Ikeji, Ebenezer Olatunde Farombi

**Affiliations:** 1 Department of Biochemistry, College of Science and Technology, Covenant University, Ota, Ogun, Nigeria; 2 Covenant Applied Informatics and Communication Africa Centre of Excellence (CApIC-ACE), Covenant University, Ota, Ogun, Nigeria; 3 Covenant University Public Health and Wellbeing Research Cluster (CUPHWERC), Covenant University, Ota, Ogun, Nigeria; 4 Department of Histopathology, Asokoro District Hospital, Abuja, Nigeria; 5 Department of Anatomic Pathology and Forensic Medicine, Nile University of Nigeria, Abuja, Nigeria; 6 Molecular Drug Metabolism and Toxicology Laboratory, Department of Biochemistry, College of Medicine, University of Ibadan, Ibadan, Nigeria

**Keywords:** age-dependent signalling, breast cancer, Nigeria, PI3K/Akt/mTOR, precision oncology, TNBC, Breast cancer subtypes, Africa

## Abstract

**Introduction:**

Breast cancer (BC) is a molecularly heterogeneous disease, and treatment outcomes are strongly shaped by subtype-specific signalling dependencies. However, there is paucity of data on the molecular pathways that drive breast cancer in African women. This study aimed to identify PI3K/AkT/mTOR pathway alterations in distinct BC age groups and subtypes to provide insights on personalized and more effective BC therapies.

**Methods:**

A total of 102 formalin-fixed paraffin-embedded malignant breast tissues from Nigerian women were collected from Abuja, Nigeria. The Expression of PI3K/AkT/mTOR pathway proteins was quantified using immunohistochemistry across age groups: Young-adults (YA: 20–39 years), Middle-Aged (MA: 40–59 years) and Older-Adults (OA: 60–79 years), also among BC subtypes: Estrogen Receptor-positive (ER+), Estrogen Receptor-positive/Progesterone Receptor-Positive (ER+/PR+), Human Epidermal Receptor 2-positive (HER2-positive), and triple-negative breast cancers (TNBC) tumors. Expression quantification was performed using IMAGE-J-WIN 64 and Statistical analysis was carried out using Graphpad Prism.

**Results:**

This study reveals profound molecular heterogeneity in Nigerian breast cancer, defined by distinct age- and subtype-specific signaling profiles. Proliferative markers PI3K and AKT peak in ER+, ER+/PR+, and TNBC subtypes, but significantly suppressed in HER2-positive tumors. A critical age-dependent transition was identified: young adults exhibit peak AKT, MDM2, and hTERT expression, whereas middle-aged patients show peak mTOR levels, and older-adult cohorts shift toward MAPK and PDK1 dominance. Genomic stability markers, such as BRCA1 and BRCA2, alongside luminal regulators like GATA3, decline progressively with both advancing age and tumor aggressiveness, reaching their lowest levels in TNBC. Conversely, the apoptotic and inflammatory landscapes evolve significantly across age-groups; executioner caspase activity is highest in younger patients, while older cohorts and TNBC subtypes demonstrate a marked enrichment of anti-apoptotic BCL2, pro-apoptotic BAX, and the inflammatory mediator NF-κB.

**Conclusion:**

Nigerian breast cancer exhibits profound molecular heterogeneity governed by both subtype and age. ER+ and ER+/PR+ tumors maintain DNA repair and apoptotic competence despite high proliferative signaling, HER2+ and TNBC subtypes display genomic instability and apoptotic evasion. Critically, the study identifies an evolving biological “engine, “ transitioning from AKT/hTERT-driven growth in young patients to a MAPK/NF-κB-dominant inflammatory profile and genomic collapse in older cohorts, necessitating age-tailored precision oncology and targeted inhibitors.

## 1 Introduction

Breast cancer (BC) is a heterogeneous disease defined by distinct molecular subtypes with divergent biology, therapeutic vulnerabilities, and outcomes (1). Large-scale multi-omic studies have established luminal, HER2-enriched, and basal-like/triple-negative breast cancer (TNBC) as major subtypes, each with characteristic driver alterations and pathway dependencies (2–4). Among these, aberrant activation of the phosphoinositide-3-kinase (PI3K)/AKT/mammalian target of rapamycin (mTOR) signaling axis represents one of the most frequent oncogenic events and is a central determinant of tumor growth, survival, metabolism, and therapy resistance (5–7).

Genetic and proteomic data converge on the clinical relevance of the PI3K/AkT/mTOR pathway. PIK3CA, encoding the p110α catalytic subunit, is mutated in roughly one-third of breast cancers, particularly enriched in hormone-receptor–positive disease, with hotspot substitutions at H1047R, E545K, and E542K (8–14). These alterations, together with upstream/downstream lesions such as PTEN loss, AkT activation, and mTOR complex dysregulation, sustain oncogenic signaling flux and predict sensitivity to pathway inhibition (10, 15, 16). Translational advances have followed, involving the PI3Kα inhibitor alpelisib significantly prolonged progression-free survival when combined with fulvestrant in PIK3CA-mutant Hormonal Receptor-positive/HER2-negative advanced breast cancer in the SOLAR-1 trial (17–22), while the mTOR inhibitor everolimus improved outcomes when added to endocrine therapy in endocrine-resistant HR-positive disease in the BOLERO-2 trial (23–29). Collectively, these data position PI3K/AkT/mTOR as a clinically actionable axis in breast cancer management.

However, most discovery pipelines and drug-development efforts have been anchored in Western cohorts, leaving critical gaps in the understanding of pathway biology in African populations (30–32). Genomic profiling of African and African-ancestry breast cancers reveals notable differences such as higher proportions of TNBC, elevated TP53 mutation burdens, and comparatively lower rates of PIK3CA mutations relative to White/European-ancestry datasets (30, 33–41). Nigeria, home to Africa’s largest population, has contributed essential insights through recent whole-genome and multi-omic studies, which identified distinct genomic subtypes (including a GATA3-enriched group) and highlighted unique patterns of copy-number alterations and mutational processes compared with The Cancer Genome Atlas (TCGA) cohorts (30, 37, 42). These population-level differences intersect with clinicopathologic realities: African and African-ancestry women are diagnosed at younger ages, experience higher rates of TNBC, and face poorer outcomes, underscoring the need for population-specific biology to inform precision therapeutics (39, 43–47).

In addition to the heterogenous molecular breast cancer landscape, transcriptional regulators that map onto subtype identity also differ. GATA3, a master driver of luminal differentiation and a favorable prognostic marker, is strongly associated with ER-positive/Luminal A tumors, while its loss promotes epithelial–mesenchymal transition–like programs and attenuates endocrine responsiveness (48–51). Conversely, TP53 alterations are hallmarks of TNBC and basal-like disease, occurring in ~60–80% of cases and shaping chemotherapy response and resistance trajectories (52–55). These canonical features suggest that the extent and pattern of PI3K/AkT/mTOR pathway activation, and its crosstalk with cell-cycle control, DNA damage response, inflammation/immune evasion, and apoptosis, may vary systematically by subtype and ancestry.

Despite these signals, systematic protein-level profiling of PI3K/AkT/mTOR components and pathway-connected regulators across Nigerian breast cancer age groups and subtypes remains scarce. Prior African cohort studies emphasize genomic differences, but few directly quantify pathway effector expression in clinically annotated, subtype-resolved Nigerian samples, limiting translation of pathway inhibitors and rational combinations to this population (35, 47). Addressing this evidence gap is essential, given that protein activation states, such as kinase abundance, downstream effector activation, and apoptosis/DNA-repair marker expression, integrate genomic context with tumor microenvironment and treatment exposure.

This study presents a comprehensive expression profiling of the PI3K/AkT/mTOR pathway and functionally linked regulators, spanning proliferative signaling (PI3K, AKT, mTOR, RAS, MAPK, MDM2, PDK1, hTERT), cell-cycle arrest (RB, E2F, FOXO, p27, GSK3β), genomic instability/DNA damage response (PTEN, BRCA1/2, p53), inflammation/immune modulation (NF-κB, GATA3), and apoptosis (BAX, BAD, BCL-2, Cyt-C, CASP3/8/9), across clinically defined ER-positive, ER/PR-positive, HER2-enriched, and TNBC tumors and age groups of Young-adults (20–39 years), Middle-aged (40–59 years) and Older-adults (60–79 years) from Nigerian patients. The study tested the hypothesis that age- and subtype-specific heterogeneity in pathway activation characterizes the Nigerian breast cancer landscape and delineate potentially actionable differences with direct implications for deploying PI3K/AkT/mTOR-directed therapies and rational combinations in African settings. By anchoring population-specific biology to therapeutic opportunity, this work seeks to advance equitable precision oncology.

## 2 Methods

### 2.1 Sample size

The sample size was calculated using STAT-CALC for sample size calculation for the population survey on epi-info software. The population size is estimated at 211, 000, 000 persons (United Nations, 2021); expected frequency of 50; acceptable margin of error = 7%; design effect = 1.0; clusters = 1; giving a minimum of 96 persons.

### 2.2 Sample collection

Formalin-fixed paraffin-embedded malignant tissues were collected from the histopathology department of Asokoro District Hospital, Abuja. The total sample size is 102. Properly stored breast tissues of Nigerian female patients collected within the period of 2023 to 2024, with complete patient data and histopathology results were included in the study. Histopathological examination of Hematoxylin and Eosin (H&E) stained sections was utilized to differentiate between malignant and benign states based on cellular morphology, architectural atypia, and invasion patterns. Tumor subtypes were further characterized through a panel of immunohistochemical (IHC) markers, including estrogen receptor, progesterone receptor, human epidermal growth factor receptor, and proliferation index protein (Ki-67). Subtyping was determined by the distinct expression profiles of these markers. Specifically, tumors were categorized based on the presence or absence of lineage-specific antigens and the proliferation index, ensuring a standardized classification for each analyzed tissue. Tumors that were clinically determined to be malignant were then used for the study based on the inclusion and exclusion criteria.

Inclusion criteria: The inclusion criteria for the selected malignant tumors were: (i) clinically conformed malignancy based on pathological results; (ii) complete patient data (iii) properly stored and sufficient tissue blocks. (iv) Malignant breast tumors from female patients only (v) Immunohistochemistry characterized tumors into breast cancer subtypes in the hospital by the pathologist.

Exclusion criteria: The exclusion criteria for the study was: (i) malignant tumors from male patients; (ii) Tumors with pathologically uncertain diagnosis (iii) tumors with incomplete patients’ data (iv) Insufficient tissue blocks.

### 2.3 Immunohistochemistry

Immunohistochemistry was used as a semi-quantitative method to determine the expression of PI3K/AkT/mTOR pathway proteins in the breast tissues of BC patients (**Supplementary Table 1**). Immunostaining was performed to determine protein expression and localization in 5 µm thick paraffin-embedded tissue sections. Breast tissue blocks were sectioned using a microtome and mounted on gelatin-coated slides (56, 57). Standardized protocols were provided by the antibody manufacturers, and several dilutions were initially tested to determine optimal staining conditions. Variations in staining performance for all antibodies were further assessed on representative breast Tumor sections. A certified pathologist reviewed and validated all antibody optimizations prior to their implementation in routine assays. Following overnight incubation in an oven to remove excess paraffin and enhance tissue adherence, sections were deparaffinized in two xylene changes and rehydrated through a series of graded ethanol washes (58). Antigen retrieval was achieved by heating rehydrated sections in a 0.01 M citrate buffer (pH 6.0) containing Triton X-100 at 80 °C for 30 minutes in a water bath, with evaporation minimized (59). After cooling to room temperature and rinsing in PBS, endogenous peroxidase activity was blocked by a 15-minute incubation period in 5% hydrogen peroxide in 70% methanol at 37 °C in the dark. Sections were then blocked with 5% bovine serum albumin for 30 minutes at room temperature, followed by 12hour incubation at 4 °C with specific primary antibodies in a humidified chamber. After washing in tris-buffered saline, sections were incubated for one hour at 25°C with horseradish peroxidase-conjugated goat anti-rabbit polyclonal secondary antibodies (Elabsciences). DAB staining was used for detection, with hematoxylin counterstaining. Finally, slides were cover-slipped with DPX and allowed to air-dry (57). For each standardized staining run, positive control tissues and negative controls (omission of primary antibody) were included to monitor staining specificity and consistency.

### 2.4 Image analysis

The histological slides were examined using a light microscope (Nikon Diaphot, USA) equipped with a digital camera (Canon D50, USA). Immunohistochemical (IHC) analysis, sections were subjected to heat-induced antigen retrieval using a citrate buffer (pH 6.0), followed by incubation with primary antibodies (**Supplementary Table 1**) overnight at 4 °C. Signal detection was achieved using a biotin-streptavidin-peroxidase complex, with 3, 3’-Diaminobenzidine (DAB) serving as the chromogen. To ensure staining normalization, all slides were processed in a single batch with identical incubation times, and negative controls (omitting the primary antibody) were included to account for non-specific background.

A total of three non-overlapping sections were analyzed per tissue sample. Tumor cells were evaluated for positive expression based on staining intensity and the percentage of immunoreactive cells, which were performed in duplicate. Positive staining was defined as the presence of visible brown DAB chromogen in ≥10% of tumor cells with an intensity ≥1+, while sections with ≤10% reactivity or no visible signal were classified as negative. Likewise, the stained sections were independently evaluated by a primary observer (MO), and a randomly selected subset was reassessed by a second blinded observer to determine inter-observer variability.

In addition to manual pathologist-based scoring, digital image analysis was performed using ImageJ-win 64 (NIH, Bethesda, MD, USA). For quantification, images were deconvoluted to separate the DAB signal from hematoxylin counterstaining, and the mean integrated density was measured across standardized regions of interest. These ImageJ-based quantitative results were cross-referenced with pathologist evaluations to ensure the robustness and reproducibility of the scoring system.

### 2.5 Statistical analysis

Protein expression levels derived from immunohistochemistry quantification were presented as percentages and mean ± standard deviation (SD). To identify significant differences in expression across the four molecular subtypes (ER+, ER+/PR+, HER2+, and TNBC) and the three age strata (Young Adult, Middle-Aged, and Older-Adult), statistical comparisons were performed using one-way analysis of variance (ANOVA). For comparisons of clinicodemographic characteristics and risk factors with molecular subtypes, Fisher’s Exact Test was employed. In instances where ANOVA indicated significance, *post-hoc* analysis was conducted using the Least Significant Difference (LSD) test to determine specific pairwise differences between groups. All statistical tests were two-tailed, and a p-value < 0.05 was considered the threshold for statistical significance. Data processing and visualization were conducted using GraphPad Prism version 10.0 (GraphPad Software, San Diego, CA, USA) and IBM SPSS Statistics for Windows, Version 23.0 (IBM Corp., Armonk, NY, USA).

## 3 Result

The analysis of PI3K/AkT/mTOR protein expression in breast tissues of female Nigerian patients with malignant breast cancer, categorized by BC subtype, revealed variations in oncogenic, cell cycle, apoptotic, DNA repair, and receptor pathways (**Supplementary Figures 1**-**7**; **Supplementary Table 2**).

### 3.1 Molecular subtype distribution and demographic correlations

**Figure 1:**
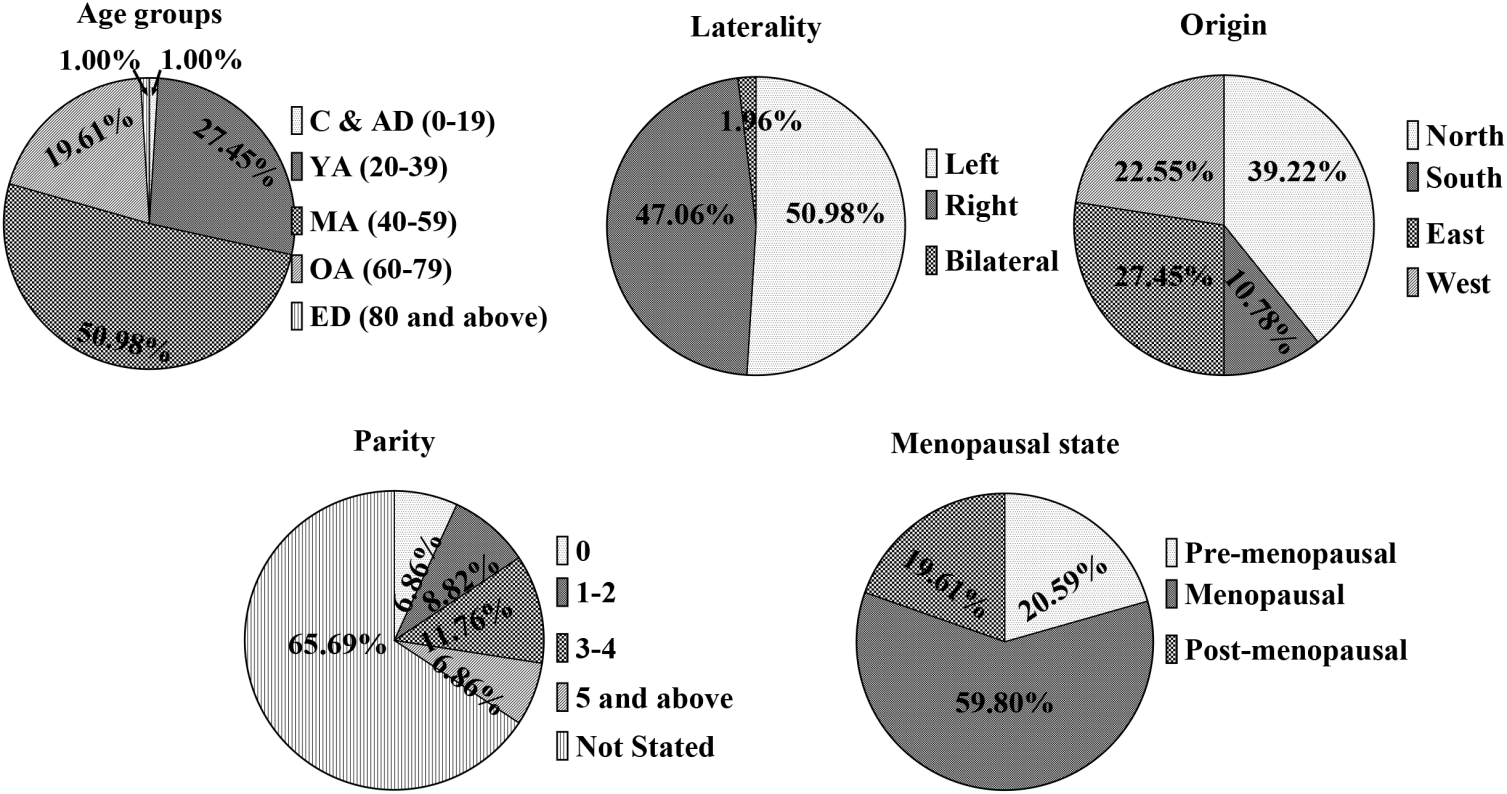
Sociodemographic characteristics of the patients. Pie charts illustrating the socio-demographic and clinical distribution of patients with malignant breast lesions (n = 102). Data are presented by age group, breast laterality, geographic origin, parity, and menopausal status.

The demographic analysis of the 102 malignant cases (**Figure 1**) reveals that the cohort was predominantly composed of middle-aged women between 40 and 59 years of age (51.0%, n = 52), followed by young adults aged 20–39 (27.5%, n = 28). Patients in the older-adult category (60–79 years) accounted for 19.6% (n = 20) of the cases, while extremes of age—including children and adolescents (1.0%, n = 1) and the elderly (1.0%, n = 1)—represented the smallest fractions of the malignant population. These data indicate that the peak incidence of malignancy in this study occurred within the fourth through sixth decades of life. Regarding breast laterality, malignant lesions demonstrated a nearly equal distribution between the left (51.0%, n = 52) and right (47.1%, n = 48) breasts. Bilateral involvement was exceptionally rare, occurring in only 2.0% of cases (n = 2), suggesting that malignancy in this population primarily presents unilaterally.

In terms of geographic origin, the regional distribution showed that the highest proportion of patients originated from the North (39.2%, n = 40), followed by the East (27.5%, n = 28) and West (22.5%, n = 23) regions. The South region contributed the smallest percentage of cases to the malignant cohort (10.8%, n = 11). The assessment of parity was partially limited by retrospective record-keeping, as a significant majority of the parity data (65.7%, n = 67) was not stated. Among the recorded data, patients with a parity of 3–4 were the most frequent (11.8%, n = 12), while those with 1–2 children (8.8%, n = 9), nulliparous women (6.9%, n = 7), and those with five or more children (6.9%, n = 7) made up the remainder of the group.

Hormonal status appeared to be a significant factor in the clinical profile, with the menopausal group forming the largest segment of the malignant cohort at 59.8% (n = 61). Pre-menopausal (20.6%, n = 21) and post-menopausal (19.6%, n = 20) women represented smaller, roughly comparable subsets. These findings highlight that the transition to menopause coincided with the highest frequency of malignant diagnoses in this study population, aligning with the peak age distribution observed.

The molecular profile of the 102 malignant cases revealed a high prevalence of Triple-Negative Breast Cancer (TNBC), accounting for 47.1% (n=48) of all cases, followed by ER+ (21.6%), ER+/PR+ (15.7%), and HER2+ (15.7%) (**Table 1**). Statistical analysis using Fisher’s Exact Test demonstrated that breast laterality, geographic region, and parity did not significantly influence the expression of molecular subtypes (p > 0.05). The “Older Adults” group (50–64 years) showed a disproportionately high percentage of TNBC (75% of cases within that age group), the overall association between age groups and molecular subtypes was not statistically significant (p = 0.551), likely due to the smaller sample sizes in the youngest and oldest strata. A significant association was observed regarding menopausal status (p = 0.041). Notably, HER2+ expression was entirely absent in the pre-menopausal group (0%) but was prominently identified in the menopausal group (23.0%). Furthermore, TNBC was the dominant subtype among post-menopausal women, representing 65.0% of that subgroup. This suggests that the hormonal milieu associated with menopausal transition may correlate with specific molecular signatures, particularly the emergence of HER2+ and TNBC phenotypes in this study population.

**Table 1:** Association of clinicodemographic characteristics and risk factors of malignant breast lesions (n = 102).

Variable	Category	ER+ (n=22)	ER+/PR+ (n=16)	HER2+ (n=16)	TNBC (n=48)	Total (n=102)	P-value
Breast location	Left	11 (21.20)	7 (13.50)	10 (19.20)	24 (46.20)	52	0.864
	Right	10 (20.80)	9 (18.80)	6 (12.50)	23 (47.90)	48	
	Bilateral	1 (50.00)	0 (0.00)	0 (0.00)	1 (50.00)	2	
Age group	Children & Adolescents	0 (0.00)	0 (0.00)	0 (0.00)	0 (0.00)	1	0.551
	Young Adults	9 (32.10)	3 (10.70)	3 (10.70)	13 (46.40)	28	
	Middle Aged	11 (21.20)	11 (21.20)	11 (21.20)	19 (36.50)	52	
	Older Aged	2 (10.00)	1 (5.00)	2 (5.00)	15 (75.00)	20	
	Elderly	0 (0.00)	1 (100.00)	0 (0.00)	(0.00)	1	
Region	North	8 (20.00)	3 (7.50)	7 (17.50)	22 (55.00)	40	0.169
	South	2 (18.20)	4 (36.40)	0 (0.00)	5 (45.50)	11	
	East	7 (25.00)	4 (14.30)	3 (10.70)	14 (50.00)	28	
	West	5 (21.70)	5 (21.70)	6 (26.10)	7 (30.40)	23	
Parity	0	3 (42.90)	0 (0.00)	1 (14.30)	3 (42.90)	7	0.316
	1–2	1 (11.10)	0 (0.00)	4 (44.40)	4 (44.40)	9	
	3–4	2 (16.70)	0 (0.00)	3 (25.00)	7 (58.30)	12	
	≥5	1 (14.30)	0 (0.00)	1 (14.30)	5 (71.40)	7	
	Not stated	15 (22.40)	16 (22.90)	7 (10.40)	29 (43.30)	67	
Menopausal state	Pre-menopausal	7 (33.30)	3 (14.30)	0 (0.00)	11 (52.40)	21	0.041*
	Menopausal	12 (19.70)	11 (18.00)	14 (23.00)	24 (39.30)	61	
	Post-menopausal	3 (15.00)	2 (10.00)	2 (10.00)	13 (65.00)	20	

*Significant at p < 0.05. Percentages are calculated row-wise.

### 3.2 Expression of proteins for sustaining proliferative signaling

**Figure 2:**
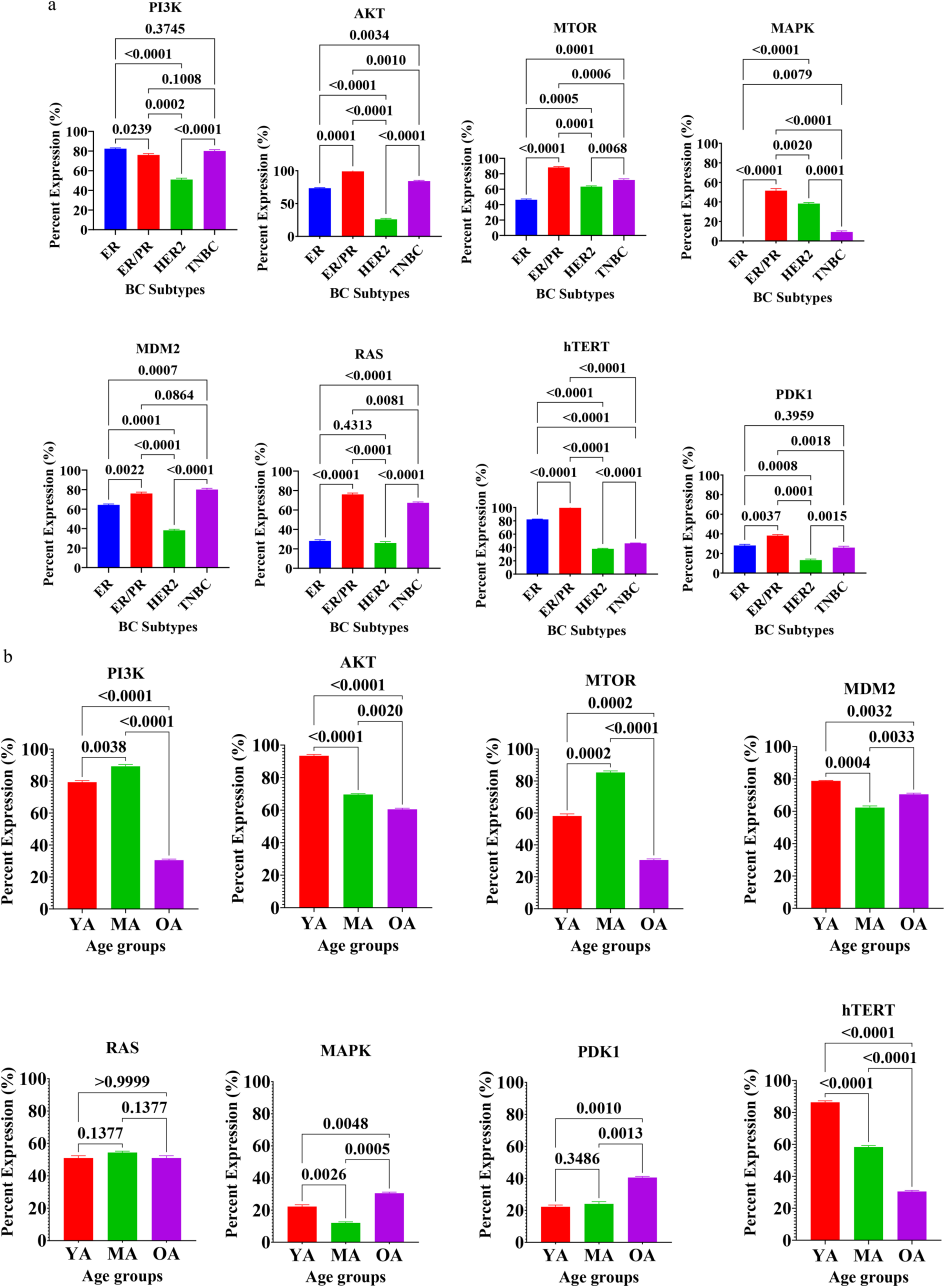
**(a)** Expression of proteins for cell proliferation in breast tissues of Nigerian breast cancer patients across BC subtypes. Values are expressed as mean ± standard deviation (SD). p < 0.0001 to p < 0.05: significant difference, p > 0.05: non- significant difference, comparison between malignant tissues across BC subtypes. ER, estrogen receptor–positive; ER/PR, dual hormone receptor–positive; HER2, HER2-enriched; TNBC, triple-negative breast cancer. **(b)** Expression of Proteins for Cell Proliferation in breast tissues of Nigerian breast cancer patients across age groups. Values are expressed as mean ± standard deviation (SD). p < 0.0001 to p < 0.05: significant difference, p > 0.05: non- significant difference, comparison between malignant tissues across age groups. YA: Young Adults (20–39 years); MA: Middle-Aged (40–59 years) and OA: Older-Aged (60–79 years).

The expression profiles of key regulatory proteins involved in sustaining proliferative signalling, specifically PI3K, AKT, mTOR, MAPK, MDM2, RAS, hTERT, and PDK1, were evaluated to characterize the molecular landscape of breast cancer in Nigerian patients (**Figures 2a, b**). Statistical analysis revealed significant heterogeneity in the expression of these markers when stratified by both molecular subtype and age group. Across the molecular subtypes (**Figure 2a**) significant differences were observed for all eight proliferative markers (**Supplementary Figures 1a**, **2**). Patients with dual hormone receptor-positive (ER+/PR+) tumors exhibited the highest mean expression levels for the majority of signaling proteins, including AKT, mTOR, MAPK, RAS, hTERT, and PDK1. In most instances, the upregulation in the ER+/PR+ tumors were statistically significant compared to the HER2-enriched and TNBC subtypes (p < 0.0001). Triple-negative breast cancer (TNBC) tissues also displayed high proliferative activity, particularly regarding PI3K and MDM2 expression, which were significantly elevated compared to the HER2-enriched group (p < 0.0001). Conversely, HER2+-enriched tumors consistently demonstrated the lowest expression levels across nearly all evaluated markers, showing significant downward variance when compared to the highly proliferative ER+/PR+ and TNBC phenotypes (p < 0.01 to p < 0.0001).

When analyzed by age group, distinct expression patterns emerged, with the notable exception of RAS, which maintained stable expression levels across all cohorts (p = ns). The young adult cohort (YA: 20–39 years) was characterized by significantly higher levels of AKT, MDM2, and hTERT by 54.83%, 12.29% and 1.86 fold respectively compared to older patients (p < 0.001). hTERT expression, in particular, showed a stark and significant inverse correlation with age, declining sharply from the youngest to the oldest age groups (p < 0.0001).In the middle-aged group (MA: 40–59 years), peak expression was observed for PI3K and mTOR. Specifically, mTOR levels in MA tissues were significantly higher than those in both the YA (p < 0.001) and older-adult (OA: 60–79 years) groups (p < 0.0001). The OA group exhibited a significant increase in MAPK and PDK1 expression compared to the MA cohort (p < 0.001 and p < 0.01, respectively). These findings suggest that while telomerase activity and AKT signaling may drive progression in younger patients, tumors in the older-adult Nigerian population may rely more heavily on the MAPK and PDK1 pathways to sustain proliferation.

### 3.3 Expression of cell cycle regulatory proteins

**Figure 3:**
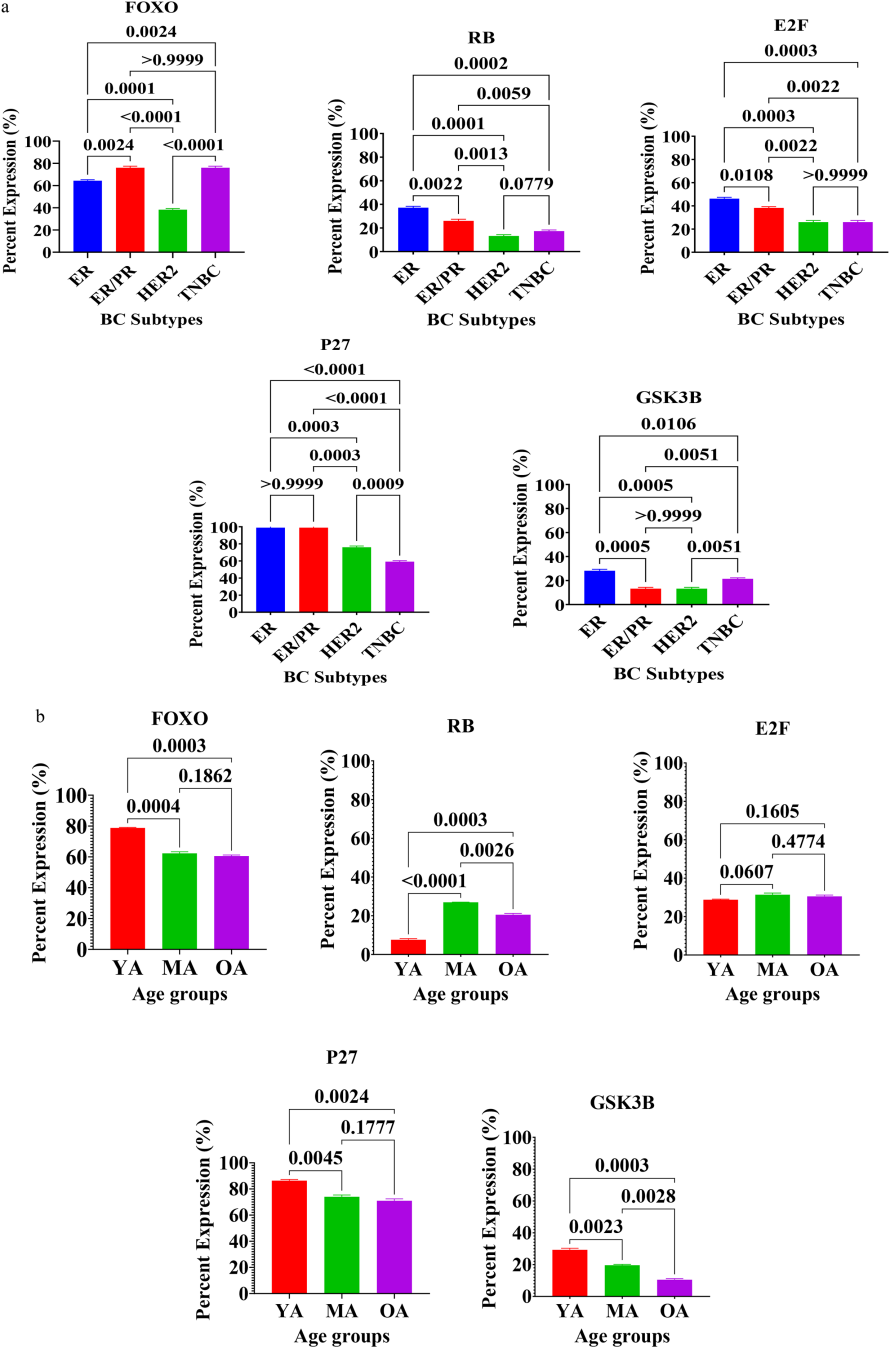
**(a)** Expression of proteins for tumor suppressor and cell cycle regulatory proteins in breast tissues of Nigerian breast cancer patients across BC subtypes. Values are expressed as mean ± standard deviation (SD). p < 0.0001 to p < 0.05: significant difference, p > 0.05: non- significant difference, comparison between malignant tissues across BC subtypes. ER, estrogen receptor–positive; ER/PR, dual hormone receptor–positive; HER2, HER2-enriched; TNBC, triple-negative breast cancer. **(b)** Expression of Proteins for Tumor suppressor and cell cycle regulatory proteins in breast tissues of Nigerian breast cancer patients across age groups. Values are expressed as mean ± standard deviation (SD). p < 0.0001 to p < 0.05: significant difference, p > 0.05: non- significant difference, comparison between malignant tissues across age groups. YA: Young Adults (20–39 years); MA: Middle-Aged (40–59 years) and OA: Older-Aged (60–79 years).

The expression profiles of tumor suppressors and cell cycle regulatory proteins, specifically FOXO, Rb, E2F, P27, and GSK3B, were analyzed in **Figures 3a, b**. Statistical analysis revealed significant variations in the expression of these regulatory nodes when stratified by molecular subtype (**Figure 3a**) and age group (**Figure 3b**).

Evaluation of cell cycle proteins across breast cancer (BC) subtypes (**Figure 3a**) demonstrated distinct regulatory environments (**Supplementary Figure 1b**). FOXO expression was significantly higher in dual hormone receptor-positive (ER+/PR+) and triple-negative breast cancer (TNBC) tumors compared to the HER2-enriched tumors (p < 0.0001). In contrast, the expression of RB and E2F was highest in the ER-positive (ER+) tumors, showing a significant decrease in HER2-enriched and TNBC tissues (p < 0.01 to p < 0.001). P27, a critical cyclin-dependent kinase inhibitor, maintained high levels in ER and ER/PR tumors but was significantly downregulated in the HER2+ and TNBC tumors (p < 0.0001). Furthermore, GSK3β expression showed significant heterogeneity, with the highest frequency observed in the ER+ tumor, followed by TNBC tumor, while ER+/PR+ and HER2+ tumor exhibited significantly lower levels (p < 0.01).

Analysis by age group (**Figure 3b**) revealed that younger patients exhibit a more significant expression of several inhibitory and regulatory proteins. The young adult cohort (YA: 20–39 years) displayed significantly higher levels of FOXO and P27 compared to the middle-aged (MA: 40–59 years) and older-adult (OA: 60–79 years) groups (p < 0.001 for FOXO; p < 0.01 for P27). Similarly, GSK3B expression was at its peak in the YA group and declined significantly with advancing age (p < 0.001 comparing YA to OA). However, RB expression showed an inverse trend, being significantly lower in young adults compared to both middle-aged (p < 0.0001) and older-adult (p < 0.001) cohorts. E2F expression remained statistically stable across all age groups, suggesting that while upstream regulators like RB vary with age, the core E2F transcriptional machinery may maintain consistent baseline expression levels in this population (p = ns).

### 3.4 Expression of proteins for regulating genomic instability/DNA damage response

**Figure 4:**
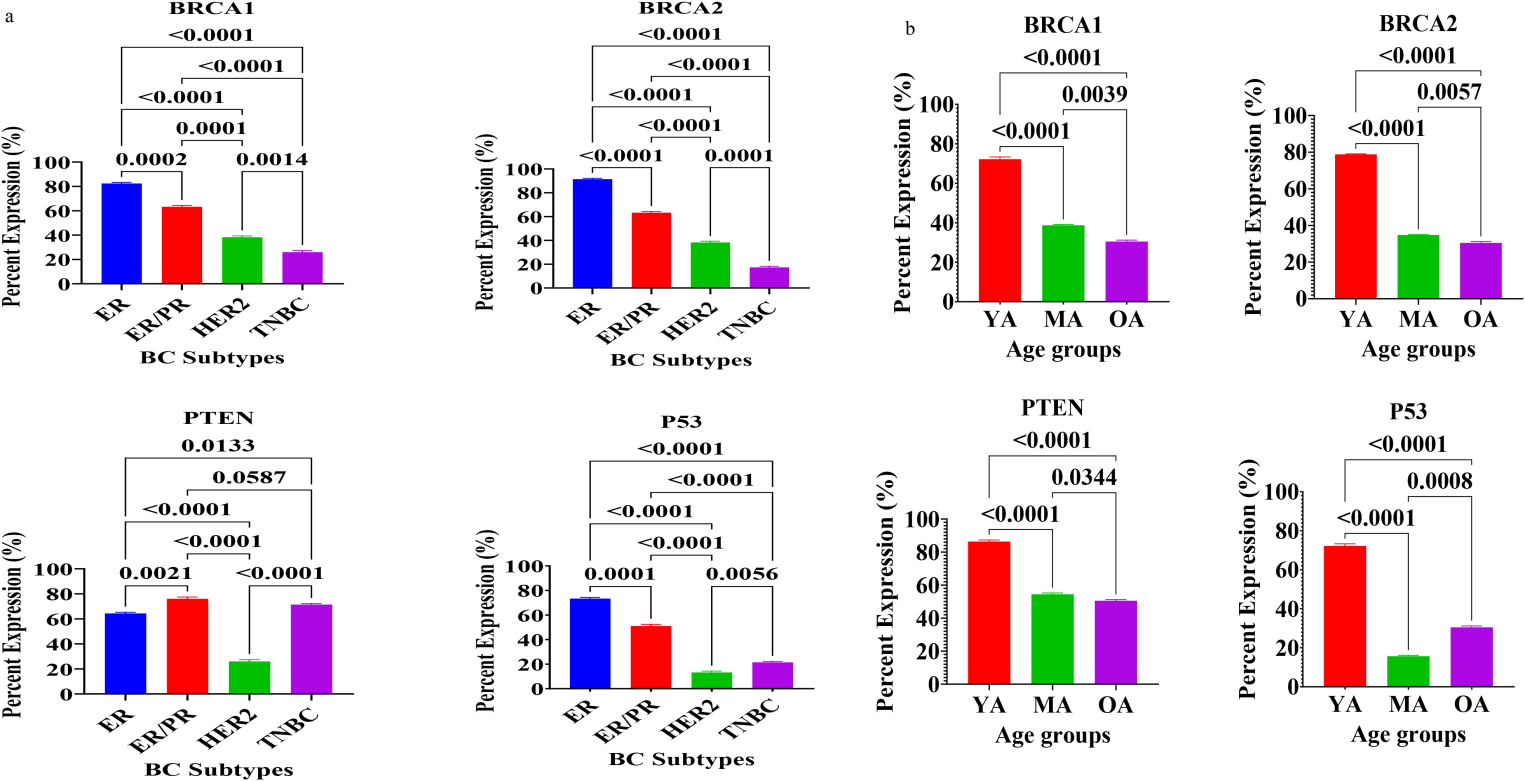
**(a)** Expression of DNA damage response regulatory proteins in breast tissues of Nigerian breast cancer patients across BC subtypes. Values are expressed as mean ± standard deviation (SD). p < 0.0001 to p < 0.05: significant difference, p > 0.05: non- significant difference, comparison between malignant tissues across BC subtypes. ER, estrogen receptor–positive; ER/PR, dual hormone receptor–positive; HER2, HER2-enriched; TNBC, triple-negative breast cancer. **(b)** Expression of DNA Damage Response Regulatory Proteins in breast tissues of Nigerian breast cancer patients across age groups. Values are expressed as mean ± standard deviation (SD). p < 0.0001 to p < 0.05: significant difference, p > 0.05: non- significant difference, comparison between malignant tissues across age groups. YA: Young Adults (20–39 years); MA: Middle-Aged (40–59 years) and OA: Older-Aged (60–79 years).

The expression profiles of key regulatory proteins involved in DNA damage response (DDR) and the maintenance of genomic stability, specifically BRCA1, BRCA2, PTEN, and p53, were analyzed (**Figure 4**). Statistical evaluations revealed significant heterogeneity in the expression of these markers when stratified by molecular subtype (**Figure 4a**) and age group (**Figure 4b**) among Nigerian breast cancer patients (**Supplementary Figure 1c**).

Quantitative analysis demonstrated that DDR protein expression is heavily influenced by the molecular subtype of the tumor. BRCA1 and BRCA2 expression levels were highest in the ER-positive tumor, showing a progressive and significant decline across the ER+/PR+, HER2+, and triple-negative breast cancer (TNBC) tumors (p < 0.0001). Notably, TNBC tissues exhibited the lowest levels of both BRCA1 and BRCA2 compared to all other subtypes (p < 0.01 and p < 0.001, respectively). Similarly, p53 expression was most expressed in ER+ tissues and significantly lower in the HER2+ and TNBC tumor (p < 0.0001). PTEN expression followed a distinct pattern; while it was significantly elevated in the ER+/PR+ and TNBC tumors, it was markedly suppressed in HER2+ tumors. The difference in PTEN expression between TNBC and HER2+ tumors was statistically significant (p < 0.0001), suggesting divergent mechanisms of genomic instability in these aggressive phenotypes.

Analysis of DDR proteins across age categories revealed a stark, age-dependent decline in the expression of these vital genomic guardians. The young adult cohort (YA: 20–39 years) exhibited significantly higher levels of BRCA1, BRCA2, PTEN, and p53 compared to middle-aged (MA: 40–59 years) and older-adult (OA: 60–79 years) patients (p < 0.0001 for all markers). The MA and OA groups showed overall lower levels of these proteins, further significant declines were noted in the OA group for BRCA1, BRCA2, and PTEN when compared to the MA cohort (p < 0.01 to p < 0.05). However, p53 expression showed a slight but significant rebound in the OA group compared to middle-aged patients (p < 0.001), though it remained significantly lower than the levels observed in the youngest patients. These findings suggest that the capacity for DNA repair and genomic maintenance may be more expressed in younger Nigerian patients, potentially reflecting a higher biological drive to counteract oncogenic stress in early-onset disease.

### 3.5 Expression of proteins for regulating tumor-promoting inflammation/avoiding immune response

**Figure 5:**
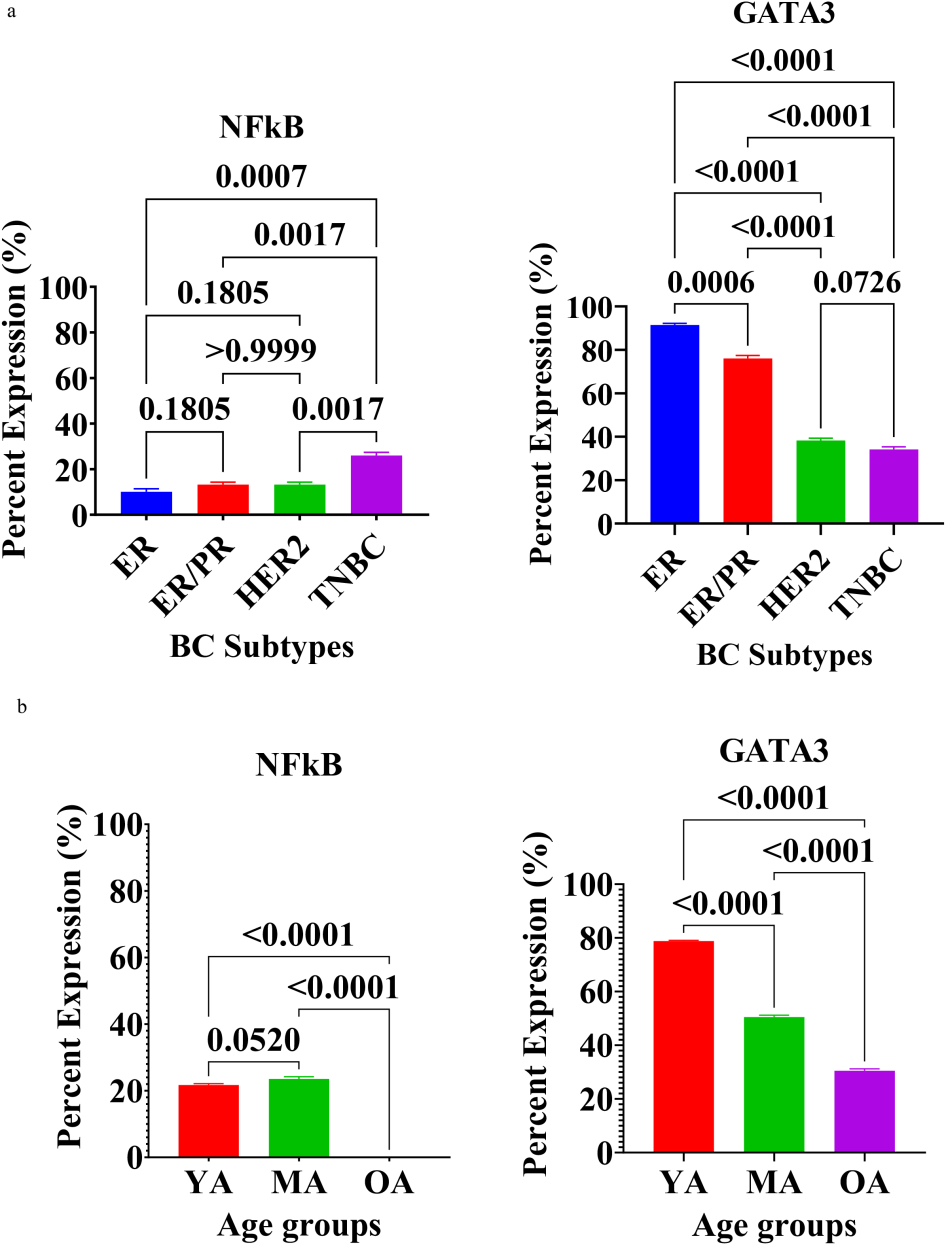
**(a)** Expression of Inflammation and Immune Response Regulatory Proteins in breast tissues of Nigerian breast cancer patients across BC subtypes. Values are expressed as mean ± standard deviation (SD). p < 0.0001 to p < 0.05: significant difference, p > 0.05: non- significant difference, comparison between malignant tissues across BC subtypes. ER, estrogen receptor–positive; ER/PR, dual hormone receptor–positive; HER2, HER2-enriched; TNBC, triple-negative breast cancer. **(b)** Expression of Inflammation and Immune Response Regulatory Proteins in breast tissues of Nigerian breast cancer patients across age groups. Values are expressed as mean ± standard deviation (SD). p < 0.0001 to p < 0.05: significant difference, p > 0.05: non- significant difference, comparison between malignant tissues across age groups. YA, Young Adults (20–39 years); MA, Middle-Aged (40–59 years); OA, Older-Aged (60–79 years).

Inflammatory and immune regulatory proteins showed distinct expression trends (**Figure 5**).

The expression of regulatory proteins associated with tumor-promoting inflammation and immune evasion, specifically Nuclear Factor kappa B (NF-κB) and GATA binding protein 3 (GATA3), was assessed across different breast cancer (BC) molecular subtypes (**Supplementary Figure 1d**) and age cohorts. The results highlight distinct immunological landscapes within the study population.

Quantitative analysis of these markers revealed significant heterogeneity depending on the molecular subtype. The expression of NF-κB, a key mediator of inflammatory signaling, was significantly higher in triple-negative breast cancer (TNBC) compared to all other subtypes (p < 0.01 to p < 0.001). The ER-positive, ER/PR-positive, and HER2-positive tumors showed comparable baseline levels of NF-κB, the TNBC tumors exhibits a more significant inflammatory microenvironment in this aggressive phenotype. Conversely, GATA3 expression—typically associated with luminal differentiation and immune regulation—demonstrated a polar opposite distribution. GATA3 levels were highest in the ER-positive group and significantly decreased across the ER/PR-positive, HER2-positve, and TNBC tumors (p < 0.001 to p < 0.0001). TNBC tumors exhibited the lowest frequency of GATA3 expression, highlighting a significant loss of this regulatory protein in hormone-independent tumors (p < 0.0001).

Analysis by age group further revealed age-dependent shifts in inflammatory and immune markers. NF-κB expression remained relatively stable between the young adult (YA) and middle-aged (MA) cohorts (p = ns); however, it showed a sharp and significant increase in the older-adult (OA: 60–79 years) group (p < 0.0001). This suggests an intensified inflammatory state in tumors arising in older Nigerian patients. In contrast, GATA3 expression exhibited a significant and progressive decline with advancing age. The YA group (20–39 years) showed the most robust GATA3 levels, which were significantly higher than those found in the MA (p < 0.0001) and OA (p < 0.0001) groups. These findings indicate that younger patients may retain more GATA3-mediated regulatory signaling, whereas the inflammatory NF-κB pathway becomes increasingly dominant in the later stages of life.

### 3.6 Expression of proteins for regulating apoptosis

**Figure 6:**
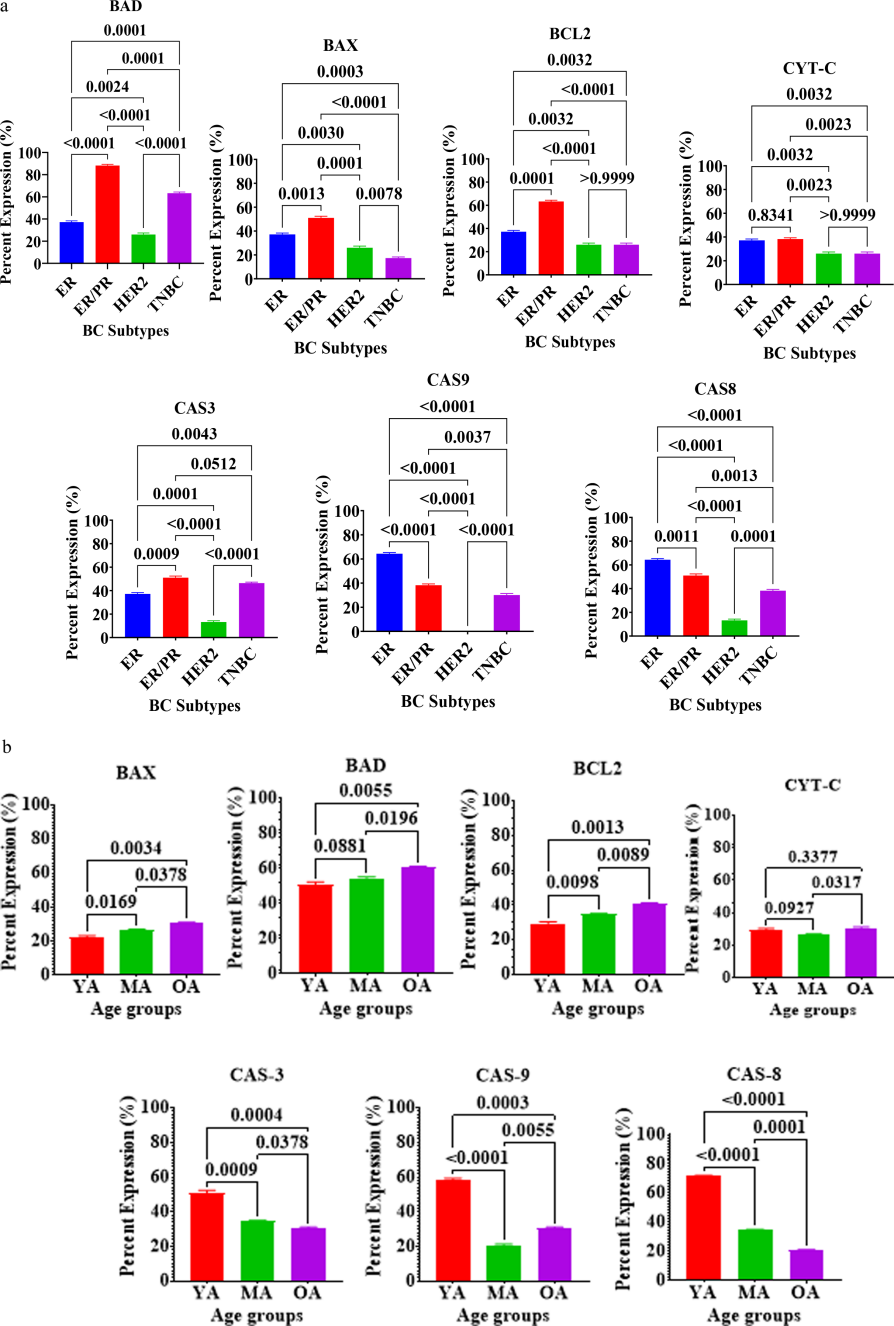
**(a)** Expression of Apoptosis Regulatory Proteins in breast tissues of Nigerian breast cancer patients across BC subtypes. Values are expressed as mean ± standard deviation (SD). p < 0.0001 to p < 0.05: significant difference, p > 0.05: non- significant difference, comparison between malignant tissues across BC subtypes. ER, estrogen receptor–positive; ER/PR, dual hormone receptor–positive; HER2, HER2-enriched; TNBC, triple-negative breast cancer. **(b)** Expression of Apoptosis Regulatory Proteins in breast tissues of Nigerian breast cancer patients across age groups. Values are expressed as mean ± standard deviation (SD). p < 0.0001 to p < 0.05: significant difference, p > 0.05: non- significant difference, comparison between malignant tissues across age groups. YA, Young Adults (20–39 years); MA, Middle-Aged (40–59 years); OA: Older-Aged (60–79 years).

The expression of key apoptotic markers—including pro-apoptotic factors (BAD, BAX, CYT-C, Caspase-3, Caspase-8, and Caspase-9) and the anti-apoptotic protein BCL2, was evaluated in **Figures 6a, b**. The data reveal significant heterogeneity in apoptotic signaling across molecular subtypes (**Supplementary Figure 1e**) and age groups.

The expression of apoptotic proteins demonstrated highly significant variations according to molecular subtype (**Figure 6a**). The dual hormone receptor-positive (ER+/PR+) tumor exhibited the highest expression levels of the pro-apoptotic markers BAD, BAX, and Caspase-3 (CAS3), as well as the anti-apoptotic marker BCL2. Specifically, the upregulation of BCL2 and BAX in the ER+/PR+ tumor was statistically significant compared to all other subtypes (p < 0.0001). Triple-negative breast cancer (TNBC) tissues also showed significant expression of BAD and CAS3, significantly exceeding the levels found in the HER2+ tumor (p < 0.0001). In contrast, the intrinsic and extrinsic pathway initiators, Caspase-9 (CAS9) and Caspase-8 (CAS8), were most prominently expressed in the ER-positive (ER+) tumor. Expression of both CAS9 and CAS8 was significantly lower in the HER2-enriched and TNBC tumor (p < 0.0001). Across nearly all evaluated apoptotic markers, HER2-enriched tumors consistently demonstrated the lowest expression, suggesting a potential suppression of apoptotic machinery in this subtype.

Analysis of apoptotic protein expression across age cohorts revealed distinct trends in cell death regulation. Pro-apoptotic markers such as BAX and BCL2 showed a significant and progressive increase with advancing age, reaching their peak in the older-adult (OA: 60–79 years) group (p < 0.05 to p < 0.01). Similarly, BAD expression was significantly higher in the OA cohort compared to the young adult (YA: 20–39 years) group (p < 0.01).Conversely, executioner and initiator caspases exhibited an inverse relationship with age. The young adult cohort displayed significantly higher levels of CAS3, CAS9, and CAS8 compared to the middle-aged (MA) and OA groups (p < 0.001 to p < 0.0001). Notably, CAS8 levels declined sharply and significantly across all three age strata (p < 0.0001). Cytochrome-c (CYT-C) levels remained relatively stable between the YA and MA groups, a significant increase was noted in the OA cohort (p < 0.05). These findings suggest that while the upstream apoptotic triggers (caspases) are more active in younger patients, older patients may shift toward a different apoptotic balance characterized by higher BCL2 and BAX levels.

## 4 Discussion

The PI3K/AkT/mTOR signaling is a central pathway implicated in most of the hallmarks of cancer. It controls cell proliferation, inflammation, protein synthesis, apoptosis, DNA damage response pathway, among others (16). This pathway is crucial for regulating cellular proliferation, survival, and metabolism, significantly impacting BC pathogenesis (7, 32), as a result, the evaluation of the expression of key proteins within this pathway across different age groups (Young adults, middle-aged and older-adult) and BC subtypes (ER-positive, ER/PR-positive, HER2-positive, and TNBC) of breast tumors is important as it provides valuable insights into the distinct mechanisms of each subtype and potential therapeutic targets.

The molecular and demographic landscape of breast cancer in Nigerian women, as delineated in this study, reveals a distinct biological profile characterized by early onset, a high prevalence of aggressive subtypes, and significant age-dependent shifts in oncogenic signaling. These findings align with emerging evidence that breast cancer in women of African ancestry follows a divergent evolutionary trajectory compared to other populations, often presenting with a higher rate of genomic instability and increased intra-tumoral heterogeneity as reported by Effiong et al. (60),; Ansari-Pour et al. (42), and Pitt et al. (61),.

### 4.1 Demographic profile and subtype prevalence

The peak incidence of malignancy in the middle-aged (40–59 years) and the high proportion of young adults (27.5%) reflect a younger age at diagnosis than typically seen in Western populations, where the median age is approximately 62 years. This “left-shift” in age is a hallmark of breast cancer in Sub-Saharan Africa and has been consistently reported in other Nigerian studies, which note median ages of diagnosis around 41–43 years (42, 60–66).

Triple-Negative Breast Cancer (TNBC) was the most prevalent subtype (47.1%), a figure significantly higher than the 15–20% global average but consistent with West African regional data where TNBC frequencies often exceed 40% as reported by (44, 46, 67–69). This study revealed a significant association between menopausal status and subtype, with HER2+ expression emerging only in menopausal/post-menopausal women (70). This contrasts with Western cohorts where HER2+ tumors are frequently associated with younger age as seen in the reports by Kim et al. (71), which reported higher HER2+ expression in patients under 40 years compared to other age groups in the white and Asian races, suggesting that the hormonal milieu and reproductive history in Nigerian women may uniquely shape the molecular signature of these tumors.

### 4.2 Sustained proliferative signaling and age-related shifts

The proliferative signaling axis is governed by several key proteins: PI3K, AKT, and mTOR form a core cascade that regulates cell growth and survival (72); MAPK (Mitogen-Activated Protein Kinase) directs cellular responses to external growth stimuli (73); hTERT (human Telomerase Reverse Transcriptase) maintains telomere length to ensure cellular immortality (74); and PDK1 serves as a master switch activating AKT (75). The findings on the PI3K/AKT/mTOR pathway underscore its central role in driving malignancy in the Nigerian population. The ER+/PR+ subtype, often considered less aggressive in Western cohorts, contrastingly exhibited the highest expression of nearly all proliferative markers, including AKT, mTOR, and hTERT. This suggests that hormone-receptor-positive disease in Nigerian patients may be driven by hyperactive kinase signaling rather than traditional estrogen-driven pathways alone, potentially explaining the poorer response to standard endocrine therapies often observed in this region (73, 76–79).

A critical finding is the age-dependent transition of signaling dominance. Younger patients (YA) showed significantly higher hTERT and AKT levels, pointing toward telomerase reactivation and AKT-driven growth as primary drivers of early-onset disease. This aligns with recent data showing that hTERT expression is a poor prognostic factor (80–82). However, the older-adult (OA) group showed a significant upregulation of MAPK and PDK1. This shift suggests that as the Nigerian patient ages, the tumor’s reliance on the canonical PI3K/AKT pathway may diminish in favor of alternative proliferative nodes, a phenomenon potentially linked to “inflammaging” and chronic systemic perturbations (83).

### 4.3 Genomic instability and DNA damage response

The DDR mechanism relies on BRCA1 and BRCA2 for the repair of double-strand DNA breaks (84), PTEN to antagonize PI3K signaling and stabilize the genome (85), and p53 to act as the “guardian of the genome” by inducing cell cycle arrest or apoptosis in response to DNA damage (86). The progressive decline of BRCA1, BRCA2, and p53 expression with age indicates a collapse of genomic maintenance in older Nigerian patients. Young adults exhibited the most enriched DDR profiles, which may be a biological response to the high mutational burden often found in early-onset African breast cancer, similar to reports by Effiong et al. (32, 60). The exceptionally low levels of BRCA1/2 in TNBC further support the “BRCAness” phenotype reported in West African women, where somatic or germline mutations in TP53 and PALB2 are common, even in the absence of a strong familial history, leading to rapid tumor progression (87, 88).

### 4.4 Inflammation and immune evasion

Tumor-promoting inflammation is largely moderated by NF-κB (Nuclear Factor kappa B), which triggers the expression of pro-inflammatory and pro-survival genes (89), while GATA3 is a master transcription factor required for the maintenance of luminal cell differentiation and the suppression of inflammatory, mesenchymal phenotypes (90). The immunological profile of this cohort was characterized by a sharp rise in NF-κB in the Older-adult group and a simultaneous loss of GATA3 across all aggressive subtypes and older age groups. The loss of GATA3 in TNBC and older patients indicates a transition toward a more dedifferentiated, aggressive phenotype that avoids immune detection. The high levels of NF-κB in TNBC support its role as a key mediator of the “tumor-promoting inflammation” hallmark, a feature increasingly recognized as a driver of racial disparities in breast cancer outcomes due to its ability to foster a pro-tumorigenic microenvironment (91).

### 4.5 Apoptotic regulation

The machinery of programmed cell death involves BAX and BAD (pro-apoptotic “executioners” that permeabilize mitochondria), BCL2 (an anti-apoptotic “protector” that prevents cell death), Cytochrome-C (which triggers the intrinsic death pathway), and the Caspase family (CAS3, 8, and 9), which act as molecular scissors to dismantle the cell (92).

The dual upregulation of pro-apoptotic BAX and anti-apoptotic BCL2 in the ER+/PR+ subtype suggests a state of “apoptotic priming, “ where cells are held in a delicate balance between survival and death (93). Younger patients appear to have more active caspase machinery (CAS3, CAS8, CAS9), indicating a high-turnover tumor environment. In contrast, older patients demonstrate a shift toward intrinsic pathway components (CYT-C, BAX), but with a concurrent rise in BCL2. This age-specific apoptotic threshold could have profound implications for the timing and selection of chemotherapy and Bcl-2 Homology 3 domain (BH3)-mimetics in Nigerian clinical practice, as older patients may require agents that specifically overcome BCL2-mediated resistance.

### 4.6 Limitations of the study

This study provides significant insights into the molecular profiling of breast cancer within the Nigerian landscape, however, several methodological and design-related boundaries must be acknowledged to contextualize the findings. A primary limitation is the reliance on immunohistochemistry (IHC) for protein expression analysis. As an expression-based study, the findings represent a proxy for pathway involvement; however, the lack of genomic validation such as Next generation sequencing (NGS) or quantitative Polymerase chain reaction (qPCR) or phospho-protein analysis (e.g., Western blotting) means that functional activation of the PI3K/Akt/mTOR axis cannot be definitively confirmed.

Consequently, interpretations regarding “pathway activation” are based on protein abundance and should be viewed as observational rather than definitive proof of signaling flux. Furthermore, the cross-sectional design of this study inherently limits the ability to make temporal or causal inferences. While the data suggests an age-dependent transition in signaling dominance—shifting from Akt-driven growth in younger patients to MAPK and PDK1 upregulation in older adults—these patterns represent a physiological snapshot of different cohorts rather than a longitudinal progression within the same individuals. Finally, many of the mechanistic inferences discussed, particularly those linking expression patterns to “inflammaging” or specific early-onset drivers, remain hypothesis-generating. Future research incorporating functional assays and longitudinal tracking is necessary to validate these perceived molecular transitions and establish causal links within this specific patient population.

## 5 Conclusion

**Figure 7:**
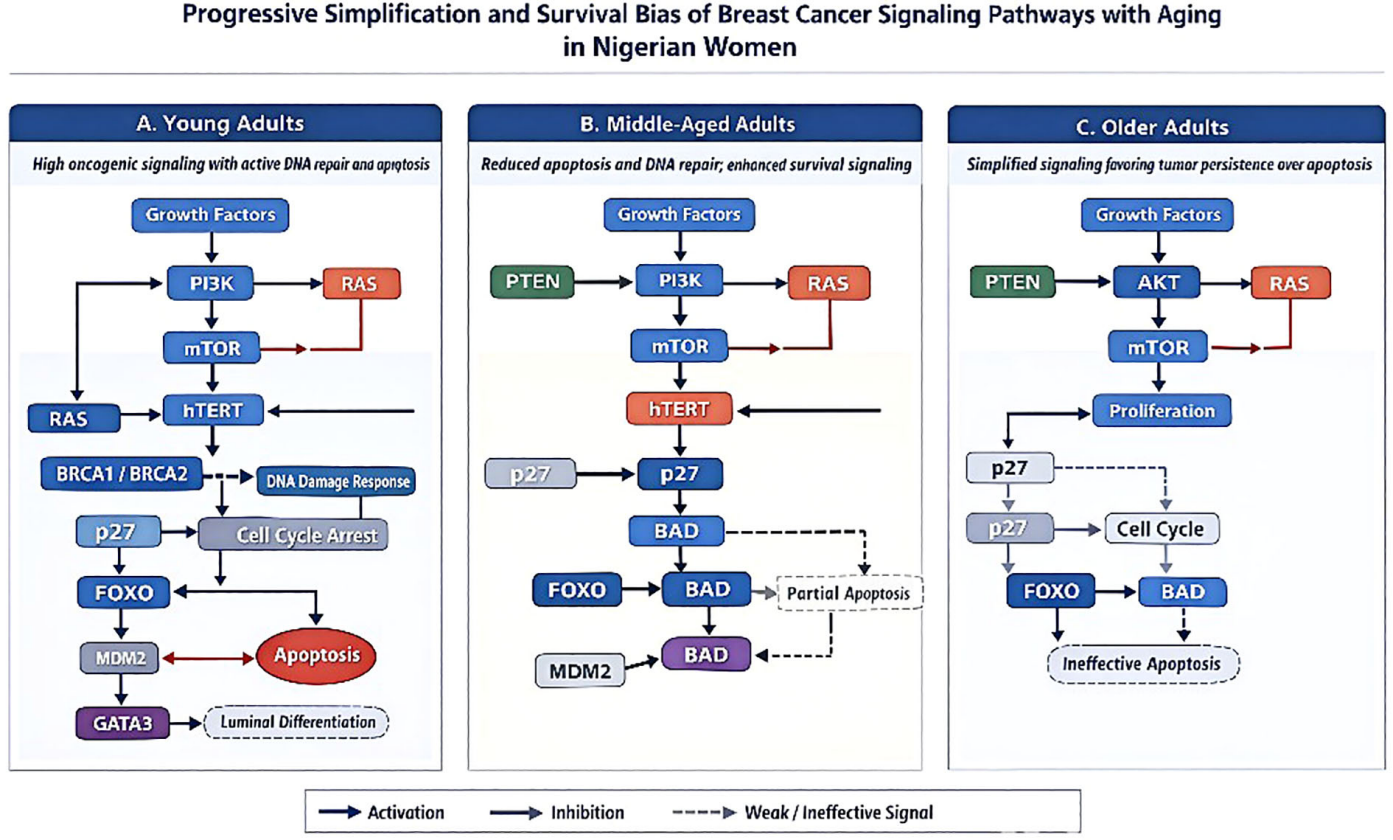
Research summary.

This study provides the first comprehensive immunohistochemical profiling of the PI3K/AkT/mTOR signaling axis across molecular subtypes in Nigerian breast cancer patients, offering novel insights into the biological heterogeneity of tumors in this understudied population. It demonstrate distinct subtype-specific expression patterns, with triple-negative and HER2-enriched cancers showing pronounced dysregulation of PI3K/AkT/mTOR pathway components compared with hormone receptor–positive tumors. The research further identifies a critical shift in molecular signatures associated with patient age. Younger adults primarily exhibit hTERT- and AKT-dominant signaling, while older patients transition to a profile dominated by MAPK and NF-κB-driven inflammation. This shift, combined with a notable loss of GATA3 and a breakdown in BRCA-mediated DNA repair—particularly in TNBC and elderly patients—points toward a state of severe genomic instability. Such biological factors likely drive the aggressive clinical progression and high mortality rates frequently observed in the region (**Figure 7**).

These results have significant translational implications: (i) they provide a rationale for incorporating PI3K/AkT/mTOR inhibitors in subtype- and age-tailored therapeutic strategies in African women; (ii) they emphasize the need to expand genomic and proteomic characterization of breast cancer in understudied populations; and (iii) they underscore the importance of integrating molecular profiling into clinical decision-making to overcome the high mortality burden in resource-limited settings. By delineating the complex heterogeneity of PI3K/AkT/mTOR pathway activation in Nigerian breast cancer, this study not only advances our understanding of tumor biology in African populations but also contributes to the global effort of developing precision oncology approaches that are equitable, context-specific, and biologically informed.

## Data Availability

The datasets presented in this study can be found in online repositories. The names of the repository/repositories and accession number(s) can be found in the article/**Supplementary Material**.

## Supplementary material

Supplementary Table 1 Details of antibodies used in immunohistochemistry procedures.

Supplementary Table 2 Expression of PI3K/AkT/mTOR proteins across breast cancer subtypes in Nigerian patients. Values (duplicates) represent number of positive cases (percentage) for each protein across breast cancer subtypes. ER+, estrogen receptor–positive; ER+/PR+, dual hormone receptor–positive; HER2+, HER2-enriched; TNBC, triple-negative breast cancer.

Supplementary Figure 1 **(a)** Representative photomicrographs of PI3K, AkT, mTOR, MAPK, MDM2, Ras, hTERT and PDK1 pathway proteins expression in malignant tissues of Nigerian breast cancer patients (Magnification = x400). The areas with brown colouring shows a positive protein expression. **(b)** Representative photomicrographs of FOXO, Rb, E2F, p27 and GSK3β pathway proteins expression in malignant tissues of Nigerian breast cancer patients (Magnification = x400). The areas with brown colouring shows a positive protein expression. **(c)** Representative photomicrographs of BRCA1, BRCA2, PTEN and p53 pathway proteins expression in malignant tissues of Nigerian breast cancer patients (Magnification = x400). The areas with brown colouring shows a positive protein expression. **(d)** Representative photomicrographs of NFkB and GATA3 pathway proteins expression in malignant tissues of Nigerian breast cancer patients (Magnification = x400). The areas with brown colouring shows a positive protein expression. **(e)** Representative photomicrographs of BAD, BCl2, BAX, CYT-C, CAS3, CAS8 and CAS 9 pathway proteins expression in malignant tissues of Nigerian breast cancer patients (Magnification = x400). The areas with brown colouring shows a positive protein expression.

Supplementary Figure 2 Expression profile of PI3K, AKT, mTOR, MDM2, hTERT, Ras, MAPK and PDK1 proteins across age groups of tumors of Nigerian breast cancer patients.

Supplementary Figure 3 Expression profile of PI3K, AKT, mTOR, MDM2, hTERT, Ras, MAPK and PDK1 proteins across BC subtypes of tumors of Nigerian breast cancer patients.

Supplementary Figure 4 Expression profile of PTEN, BRCA1, BRCA2, p53, p27, FOXO, GSK3β, Rb, E2F proteins across age groups of tumors of Nigerian breast cancer patients.

Supplementary Figure 5 Expression profile of PTEN, BRCA1, BRCA2, p53, p27, FOXO, GSK3β, Rb, E2F proteins across breast cancer subtypes of tumors of Nigerian breast cancer patients.

Supplementary Figure 6 Expression profile of Cas 8, Cas 9, Cas 3, BAD, Cyt-C, BAX, BCL-2, GATA3 and NFkB proteins across breast cancer subtypes of tumors of Nigerian breast cancer patients.

Supplementary Figure 7 Expression profile of Cas 8, Cas 9, Cas 3, BAD, Cyt-C, BAX, BCL-2, GATA3 and NFkB proteins across age groups of tumors of Nigerian breast cancer patients.
