# Supplementary material for: PI3K/Akt/mTOR pathway expression profiling reveals age- and subtype-specific molecular heterogeneity in the Nigerian breast cancer landscape
Source: Front Oncol. 2026 Mar 11;16:1766066. doi: 10.3389/fonc.2026.1766066 (PMC13012984; doi:10.3389/fonc.2026.1766066)
Supplement: Supplementary Table 1 — Details of antibodies used in immunohistochemistry procedures. [file DataSheet1.docx]

Supplementary Table 2.1: Details of antibodies used in immunohistochemistry procedures

| S/N | Antibodies |  | Dilution factor | Catalogue Number |
| --- | --- | --- | --- | --- |
| **A** | **Genes for sustaining proliferative signaling** |  |  |  |
| 1 | Phosphoinositide 3-kinase | PI3K | 1: 50 - 1:200 | E-AB-91487 |
| 2 | Protein Kinase B | AKT | 1:100 - 1:300 | E-AB-30467 |
| 3 | Mammalian Target of Rapamycin | mTOR | 1:50 - 1:200 | E-AB-15789 |
| 4 | Rat Sarcoma | Ras | 1:100 - 1:300 | E-AB-32152 |
| 5 | Mitogen-Activated Protein Kinase | MAPK/p38 | 1:100 - 1:300 | E-AB-21027 |
| 6 | Mouse Double Minute 2 | MDM2 | 1:100 - 1:300 | E-AB-31995 |
| 7 | Phosphoinositide-Dependent Kinase 1 | PDK1 | 1:100 - 1:300 | E-AB-32535 |
| 8 | human Telomerase Reverse Transcriptase | hTERT | 1:50 - 1:200 | E-AB-12901 |
| **B** | **Genes for cell cycle arrest** |  |  |  |
| 9 | Retinoblastoma | Rb | 1:100 - 1:300 | E-AB-14899 |
| 10 | Early Region 2 Binding Factor | E2F | 1:50 - 1:100 | E-AB-40147 |
| 11 | FOrkhead boX O | FOXO | 1:300-1:1000 | E-AB-70144 |
| 12 | cyclin-dependent kinase inhibitor 1B | p27 | 1:50 - 1:200 | E-AB-10569 |
| 13 | Glycogen synthase kinase 3 beta | GSK3β | 1:100 - 1:300 | E-AB-31629 |
| **C** | **Genes for regulating genomic instability/DNA damage response** |  |  |  |
| 14 | Phosphatase and TENsin homolog deleted on chromosome 10 | PTEN | 1:20 - 1:100 | E-AB-19312 |
| 15 | BReast CAncer type 1 susceptibility protein | BRCA1 | 1:100 - 1:200 | E-AB-40282 |
| 16 | BReast CAncer type 2 susceptibility protein | BRCA2 | 1:100 - 1:200 | E-AB-40288 |
| 17 | Tumor related Protein 53 | p53 | 1:100 - 1:300 | E-AB-32469 |
| **D** | **Genes for regulating tumor promoting inflammation/avoiding immune response** |  |  |  |
| 18 | Nuclear Factor Kappa B | NF-kB | 1:50-1:200 | E-AB-60843 |
| 19 | GATA binding protein 3 | GATA3 | 1:100 - 1:300 | E-AB-19493 |
| **E** | **Genes for regulating apoptosis** |  |  |  |
| 20 | BCL2-Associated X protein | BAX | 1:100-200 | E-AB-22128 |
| 21 | BCL2-Associated agonist of cell Death | BAD | 1:50 - 1:200 | E-AB-13813 |
| 22 | B-cell lymphoma 2 protein | BCl-2 | 1:50-1:100 | E-AB-60012 |
| 23 | Cytochrome C | Cyt-C | 1:50 - 1:200 | D-AB-10419L |
| 24 | Caspase 3 | CAS-3 | 1:50 -1:200 | E-AB-13815 |
| 25 | Caspase 9 | CAS-9 | 1:100 - 1:300 | E-AB-30760 |
| 26 | Caspase 8 | CAS-8 | 1:30 - 1:150 | E-AB-19664 |
|  | Peroxidase HRP-conjugated | HRP-DAB | 1:400-4000 | E-AB-1003 |

Supplementary Table 3.1: Expression of PI3K/AkT/mTOR proteins across breast cancer subtypes in Nigerian patients

| **Protein** | **ER (n=22)** | **ER/PR (n=16)** | **HER2 (n=16)** | **TNBC (n=48)** | **Total (N=102)** |
| --- | --- | --- | --- | --- | --- |
| **PI3K** | 18 (81.8) | 12 (75.0) | 8 (50.0) | 38 (79.2) | 76 (74.5) |
| **MTOR** | 10 (45.5) | 14 (87.5) | 10 (62.5) | 34 (70.8) | 68 (66.7) |
| **AKT** | 16 (72.7) | 16 (100) | 4 (25.0) | 40 (83.3) | 76 (74.5) |
| **FOXO** | 14 (63.6) | 12 (75.0) | 6 (37.5) | 36 (75.0) | 68 (66.7) |
| **MDM2** | 14 (63.6) | 12 (75.0) | 6 (37.5) | 38 (79.2) | 70 (68.6) |
| **RAS** | 6 (27.3) | 12 (75.0) | 4 (25.0) | 32 (66.7) | 54 (52.9) |
| **PTEN** | 14 (63.6) | 12 (75.0) | 4 (25.0) | 34 (70.8) | 64 (62.7) |
| **BAD** | 8 (36.4) | 14 (87.5) | 4 (25.0) | 30 (62.5) | 56 (54.9) |
| **P27** | 22 (100) | 16 (100) | 12 (75.0) | 28 (58.3) | 78 (76.5) |
| **BRCA1** | 18 (81.8) | 10 (62.5) | 6 (37.5) | 12 (25.0) | 46 (45.1) |
| **BRCA2** | 20 (90.9) | 10 (62.5) | 6 (37.5) | 8 (16.7) | 44 (43.1) |
| **GATA3** | 20 (90.9) | 12 (75.0) | 6 (37.5) | 16 (33.3) | 54 (52.9) |
| **HTERT** | 18 (81.8) | 16 (100) | 6 (37.5) | 22 (45.8) | 62 (60.8) |
| **P53** | 16 (72.7) | 8 (50.0) | 2 (12.5) | 10 (20.8) | 36 (35.3) |
| **CAS 3** | 8 (36.4) | 8 (50.0) | 2 (12.5) | 22 (45.8) | 40 (39.2) |
| **CAS 8** | 14 (63.6) | 8 (50.0) | 2 (12.5) | 18 (37.5) | 42 (41.2) |
| **CAS 9** | 14 (63.6) | 6 (37.5) | 0 (0.0) | 14 (29.2) | 34 (33.3) |
| **MAPK** | 0 (0.0) | 8 (50.0) | 6 (37.5) | 4 (8.3) | 18 (17.6) |
| **NFKB** | 2 (9.1) | 2 (12.5) | 2 (12.5) | 12 (25.0) | 18 (17.6) |
| **GSK3B** | 6 (27.3) | 2 (12.5) | 2 (12.5) | 10 (20.8) | 20 (19.6) |
| **PDK1** | 6 (27.3) | 6 (37.5) | 2 (12.5) | 12 (25.0) | 26 (25.5) |
| **BCL-2** | 8 (36.4) | 10 (62.5) | 4 (25.0) | 12 (25.0) | 34 (33.3) |
| **BAX** | 8 (36.4) | 8 (50.0) | 4 (25.0) | 8 (16.7) | 28 (27.5) |
| **CYT-C** | 8 (36.4) | 6 (37.5) | 4 (25.0) | 12 (25.0) | 30 (29.4) |
| **E2F** | 10 (45.5) | 6 (37.5) | 4 (25.0) | 12 (25.0) | 32 (31.4) |
| **RB** | 8 (36.4) | 4 (25.0) | 2 (12.5) | 8 (16.7) | 22 (21.6) |

Values (duplicates) represent number of positive cases (percentage) for each protein across breast cancer subtypes. ER+ = estrogen receptor–positive; ER+/PR+ = dual hormone receptor–positive; HER2+ = HER2-enriched; TNBC = triple-negative breast cancer.


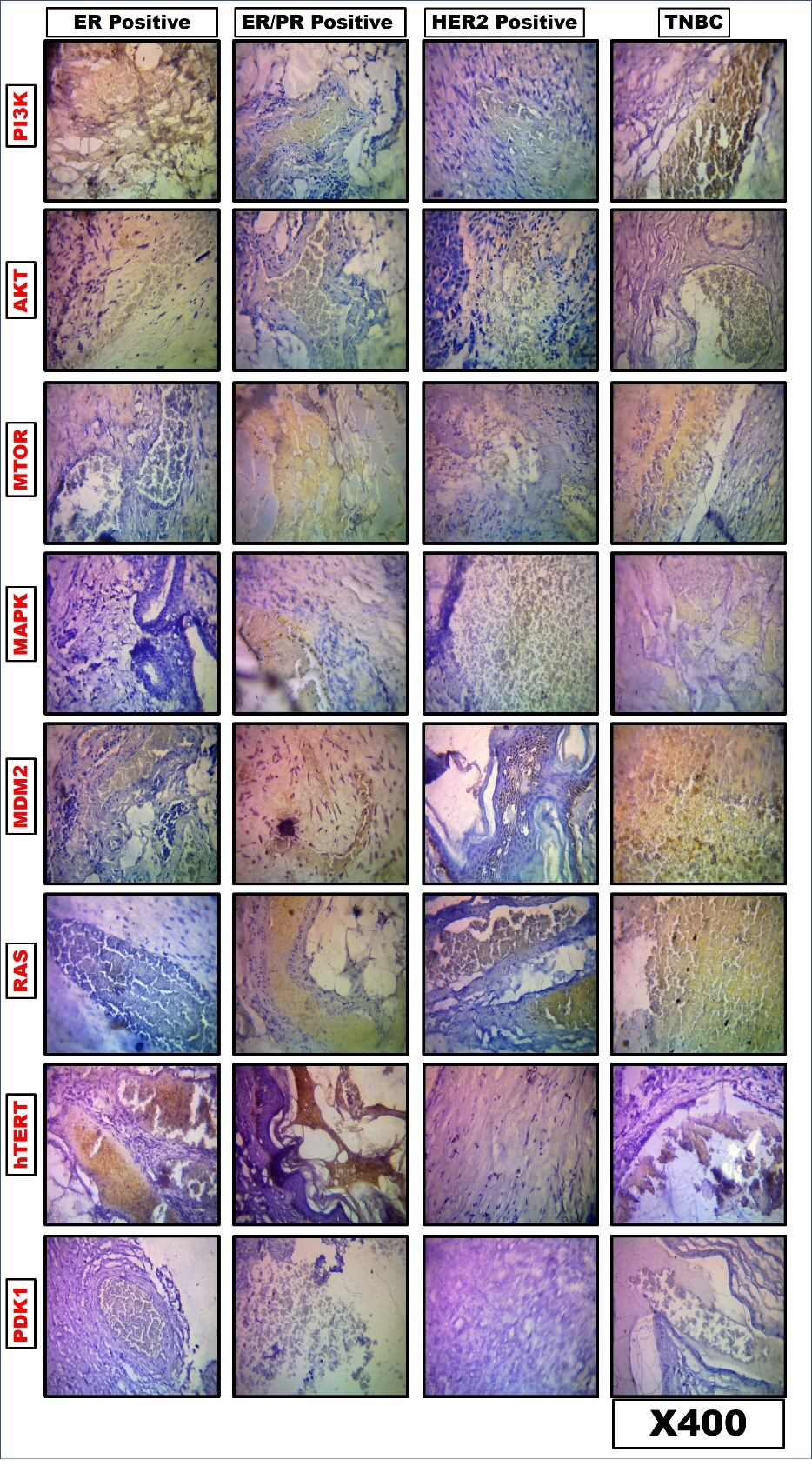


Supplementary Figure 1a: Representative photomicrographs of PI3K, AkT, mTOR, MAPK, MDM2, Ras, hTERT and PDK1 pathway proteins expression in malignant tissues of Nigerian breast cancer patients (Magnification = x400). The areas with brown colouring shows a positive protein expression.


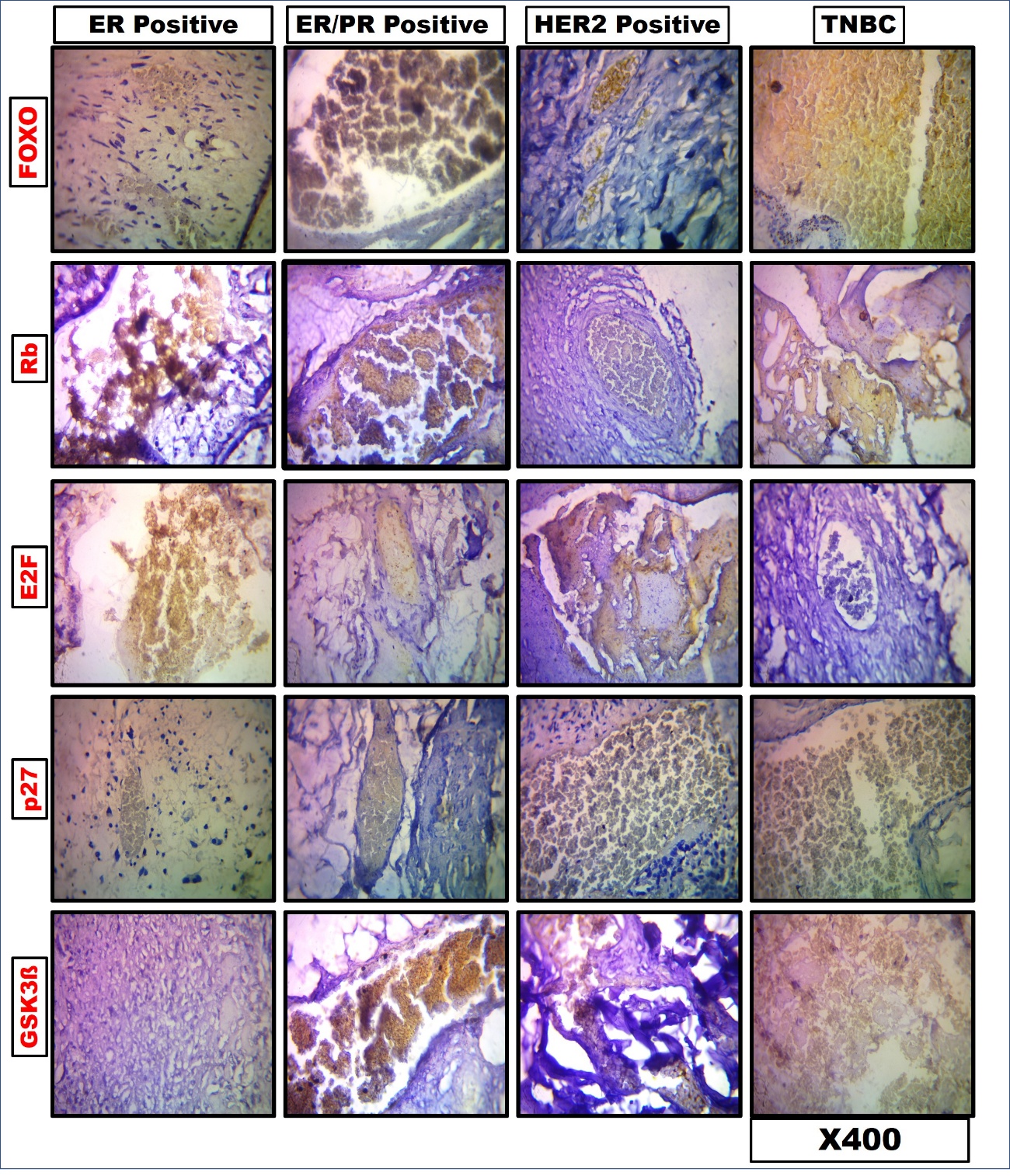


Supplementary Figure 1b: Representative photomicrographs of FOXO, Rb, E2F, p27 and GSK3β pathway proteins expression in malignant tissues of Nigerian breast cancer patients (Magnification = x400). The areas with brown colouring shows a positive protein expression.


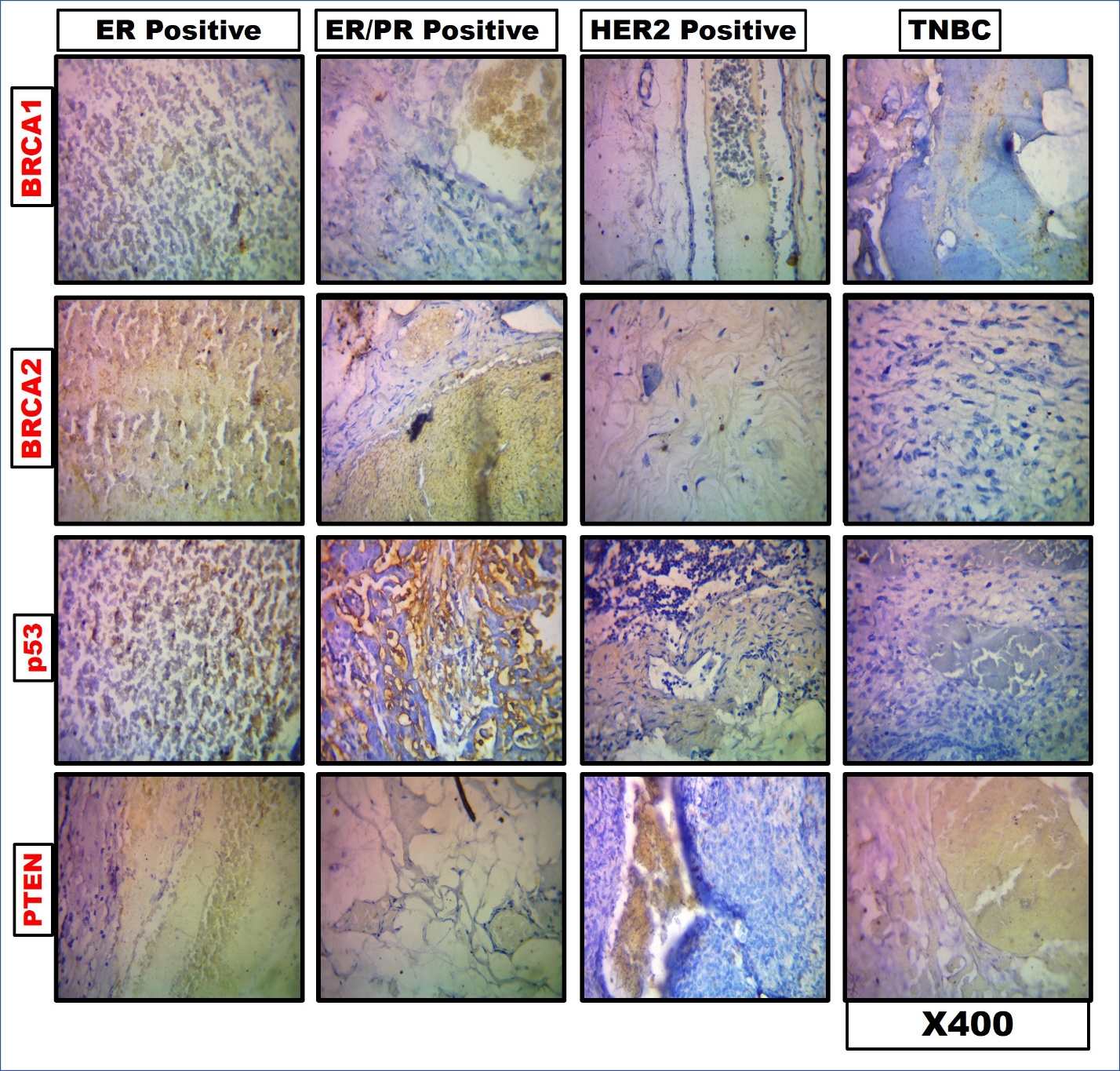


Supplementary Figure 1c: Representative photomicrographs of BRCA1, BRCA2, PTEN and p53 pathway proteins expression in malignant tissues of Nigerian breast cancer patients (Magnification = x400). The areas with brown colouring shows a positive protein expression.


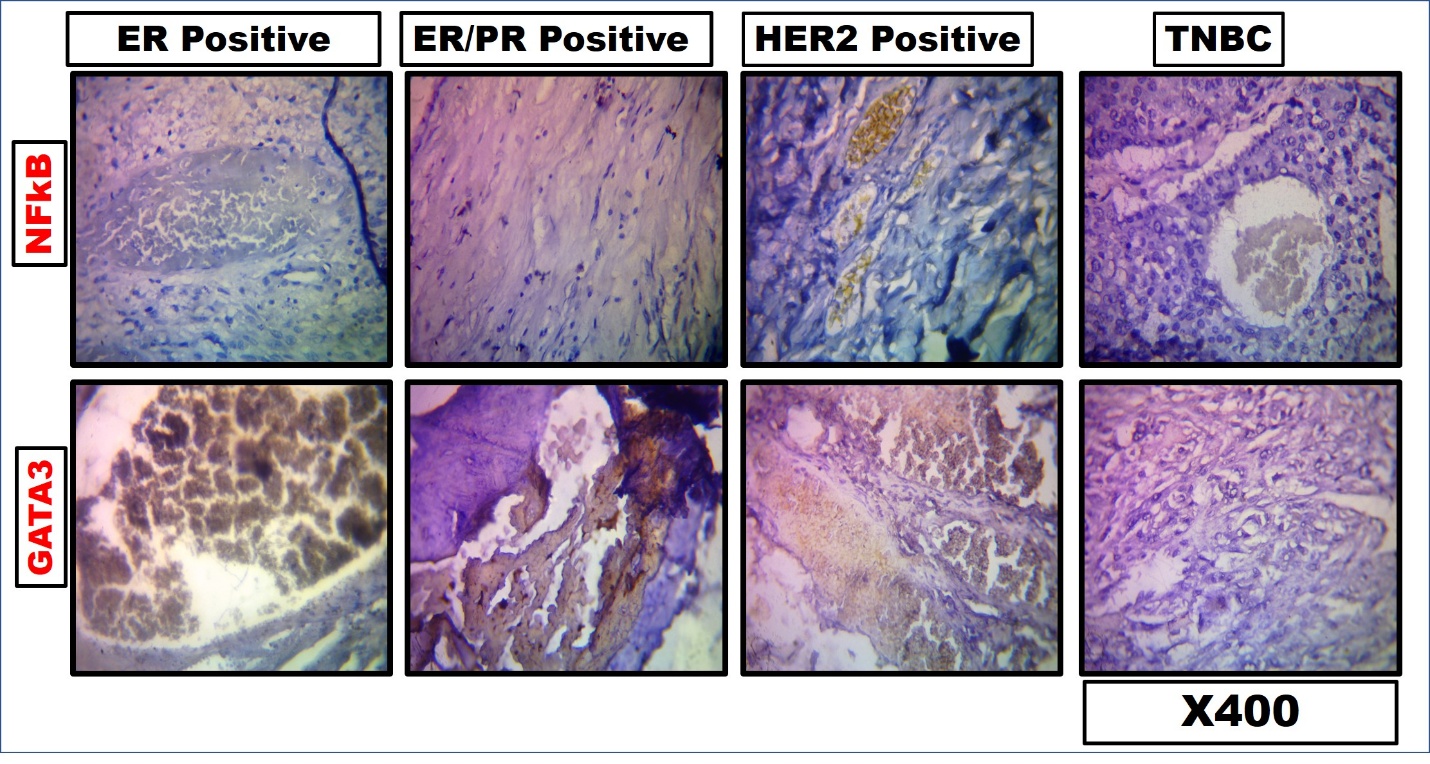


Supplementary Figure 1d: Representative photomicrographs of NFkB and GATA3 pathway proteins expression in malignant tissues of Nigerian breast cancer patients (Magnification = x400). The areas with brown colouring shows a positive protein expression.


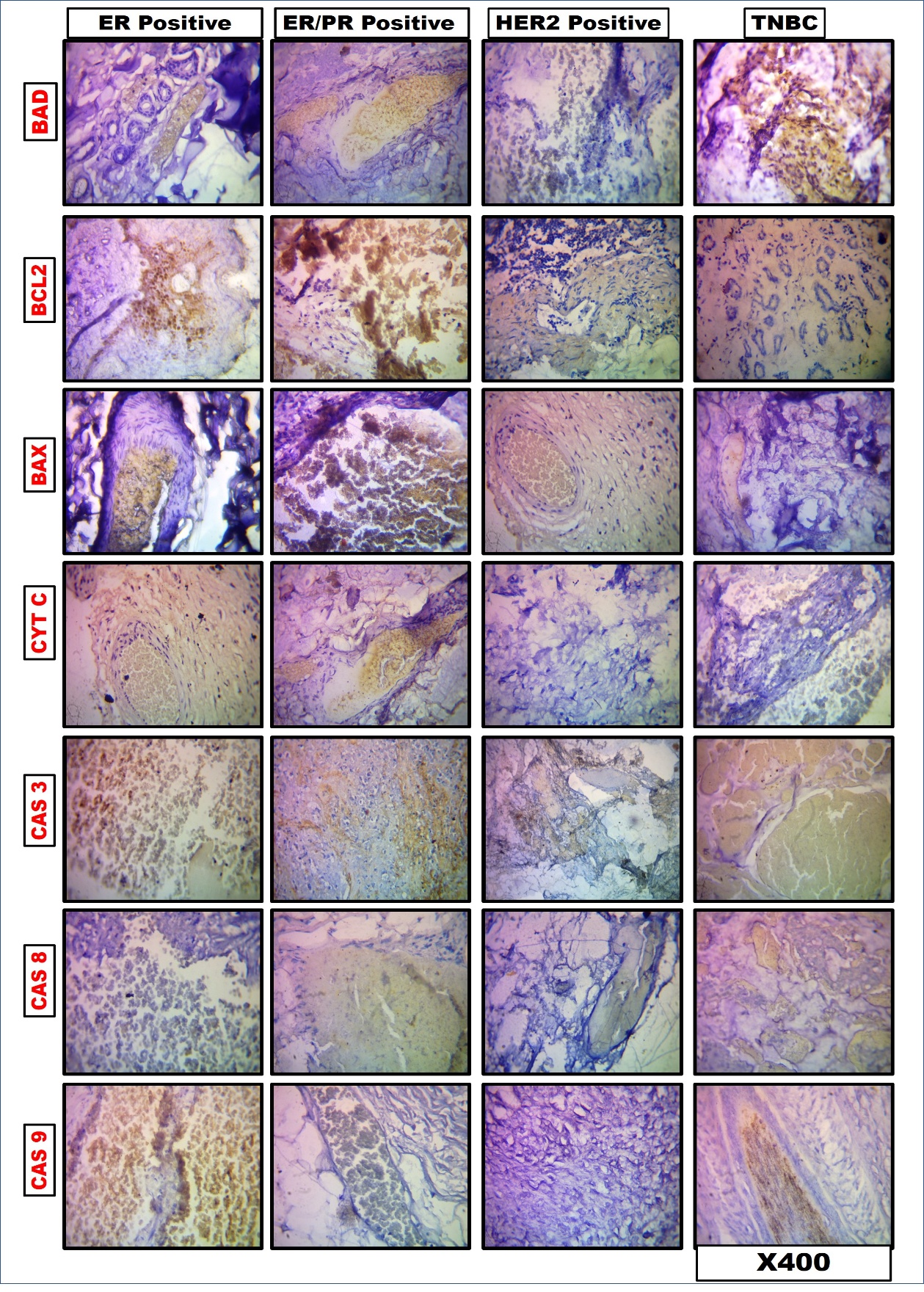


Supplementary Figure 1e: Representative photomicrographs of BAD, BCl2, BAX, CYT-C, CAS3, CAS8 and CAS 9 pathway proteins expression in malignant tissues of Nigerian breast cancer patients (Magnification = x400). The areas with brown colouring shows a positive protein expression.


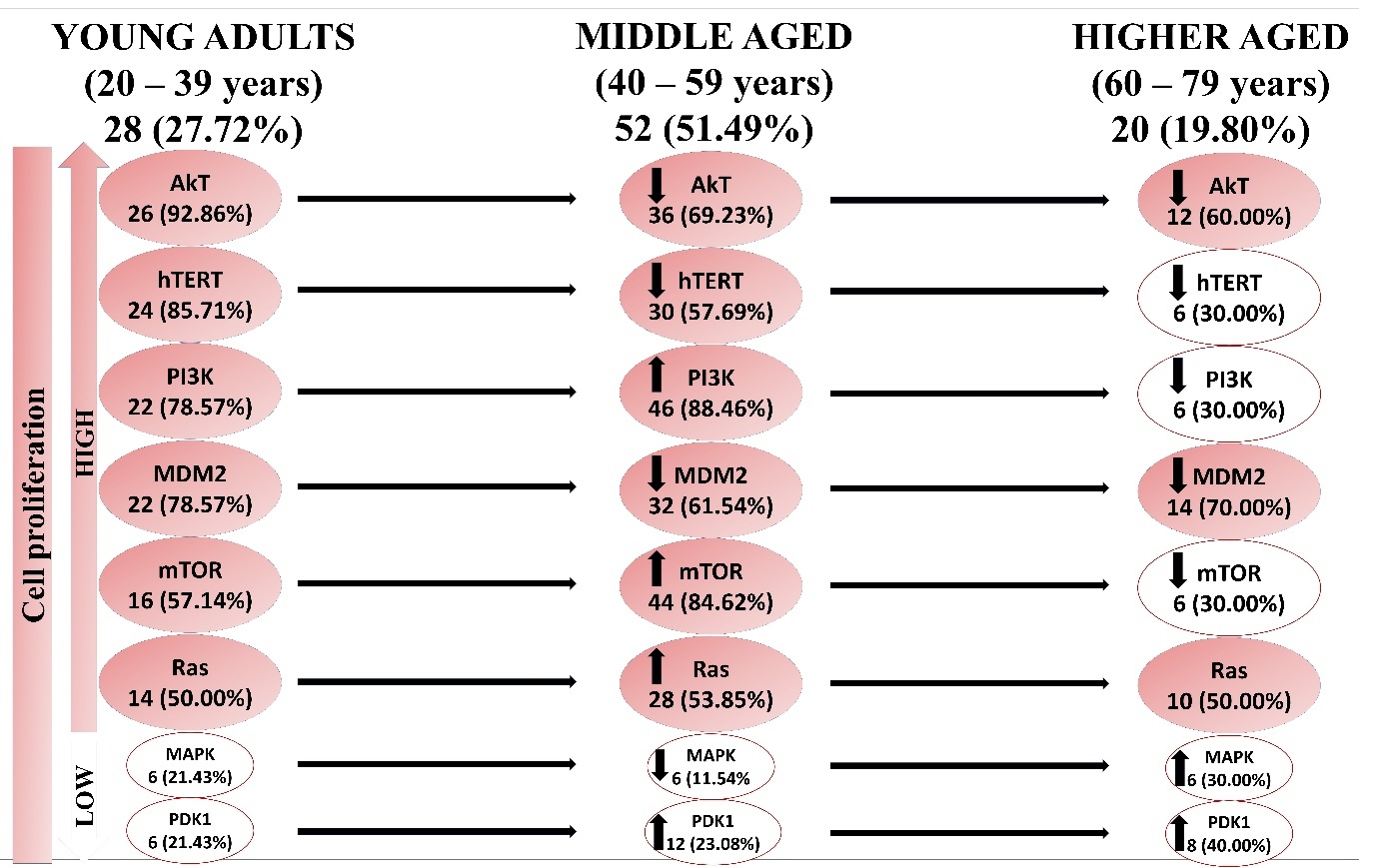


Supplementary Figure 2: Expression profile of PI3K, AKT, mTOR, MDM2, hTERT, Ras, MAPK and PDK1 proteins across age groups of tumors of Nigerian breast cancer patients.


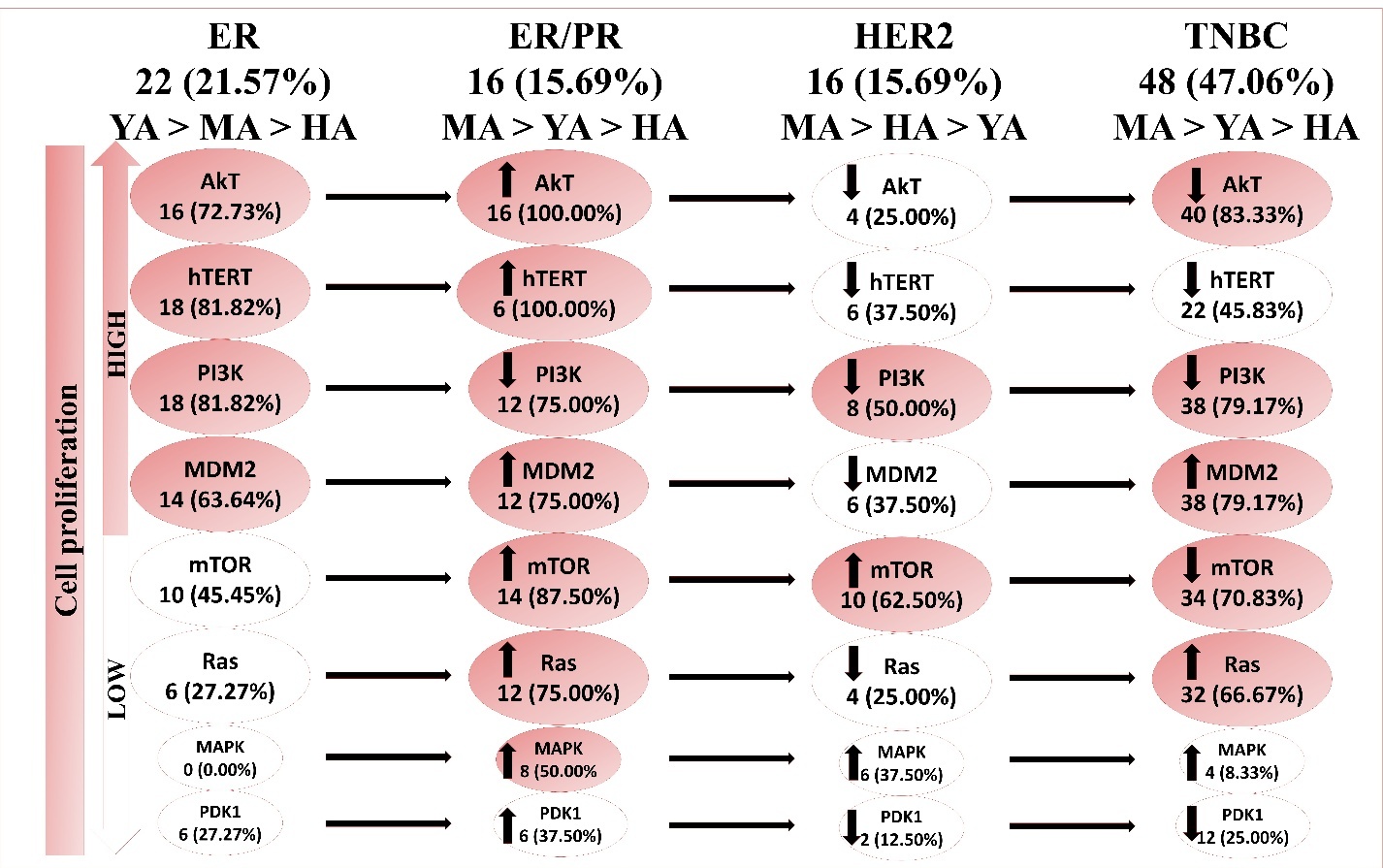


Supplementary Figure 3: Expression profile of PI3K, AKT, mTOR, MDM2, hTERT, Ras, MAPK and PDK1 proteins across BC subtypes of tumors of Nigerian breast cancer patients


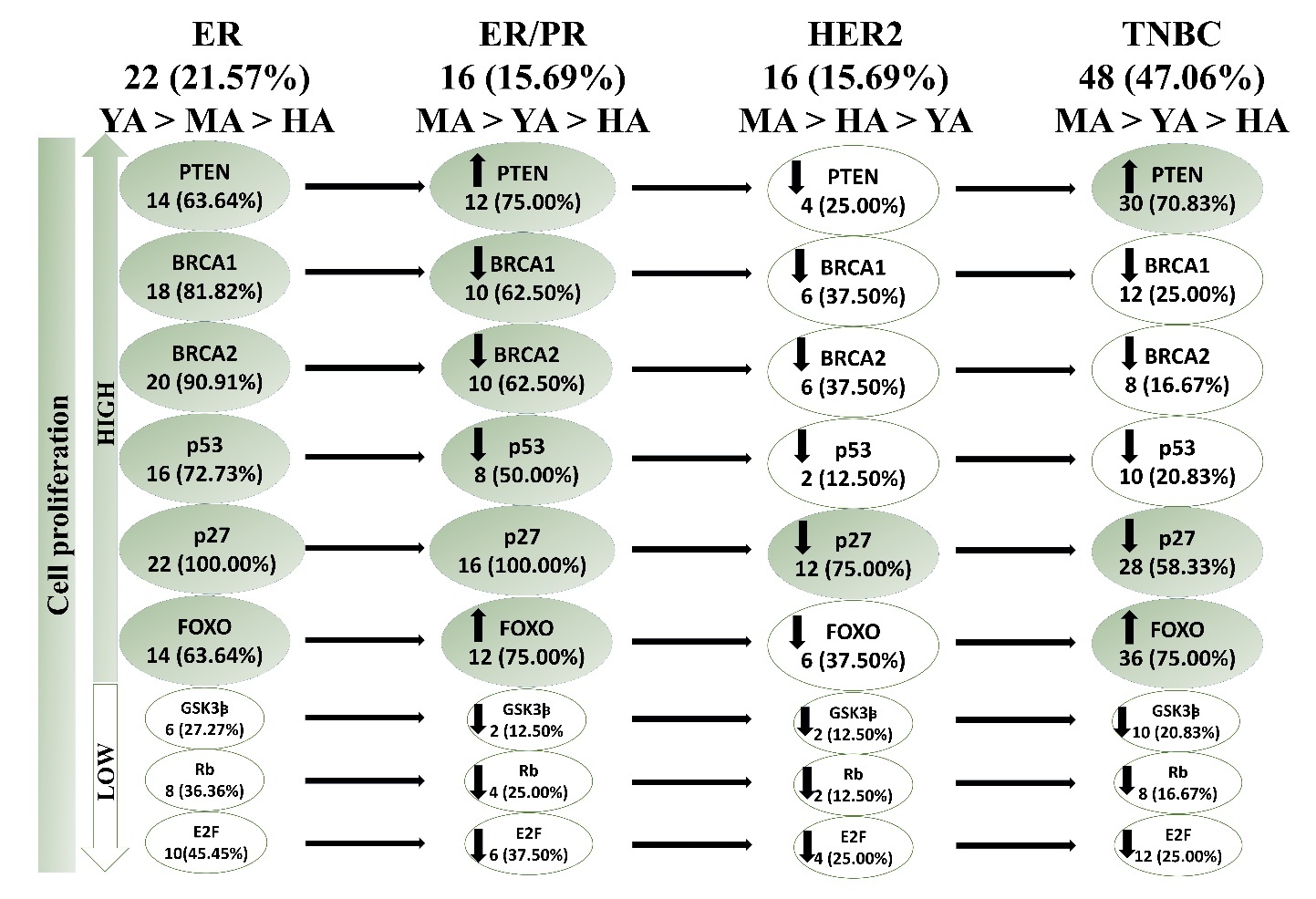


Supplementary Figure 4: Expression profile of PTEN, BRCA1, BRCA2, p53, p27, FOXO, GSK3β, Rb, E2F proteins across age groups of tumors of Nigerian breast cancer patients


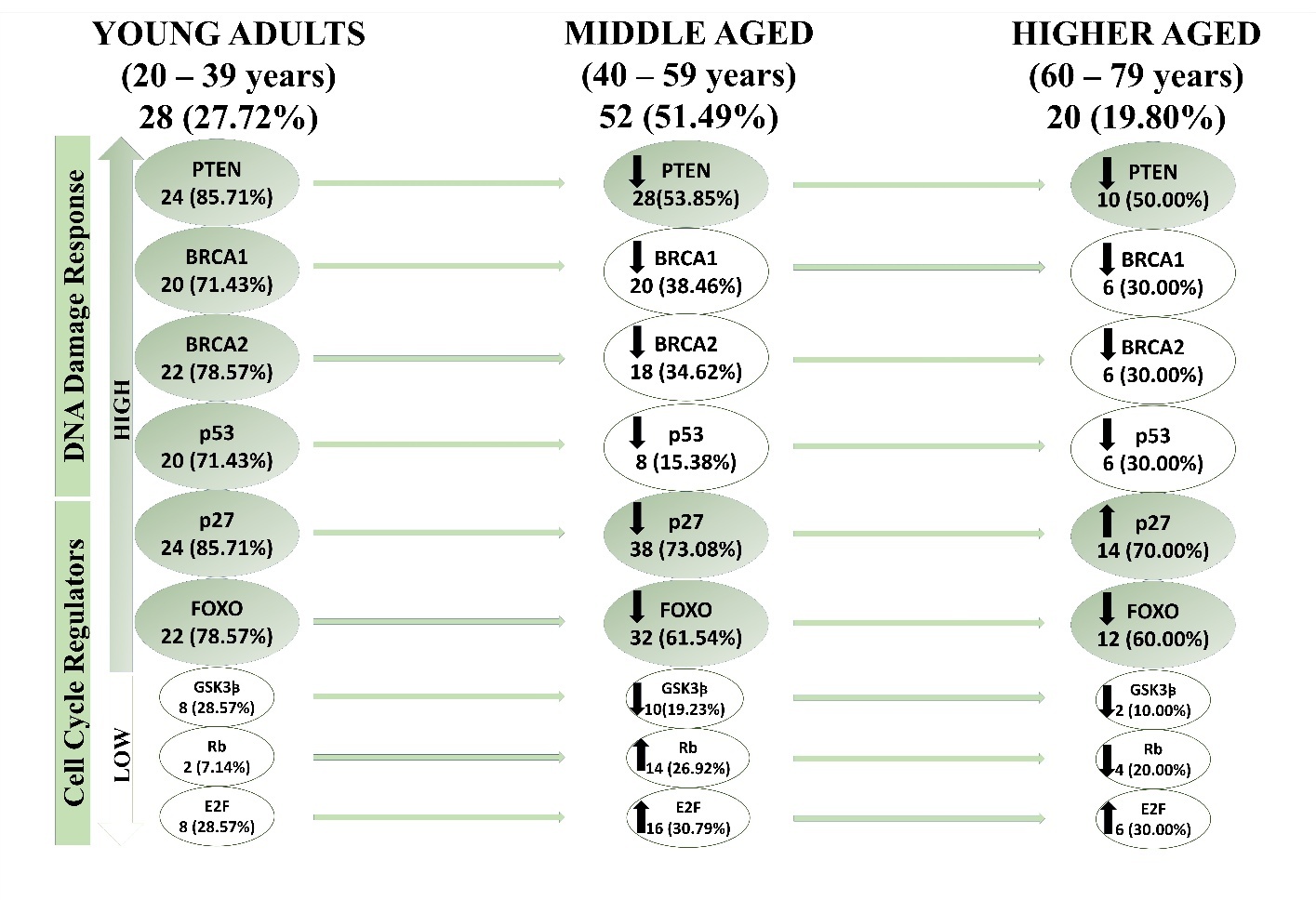
Supplementary Figure 5: Expression profile of PTEN, BRCA1, BRCA2, p53, p27, FOXO, GSK3β, Rb, E2F proteins across breast cancer subtypes of tumors of Nigerian breast cancer patients


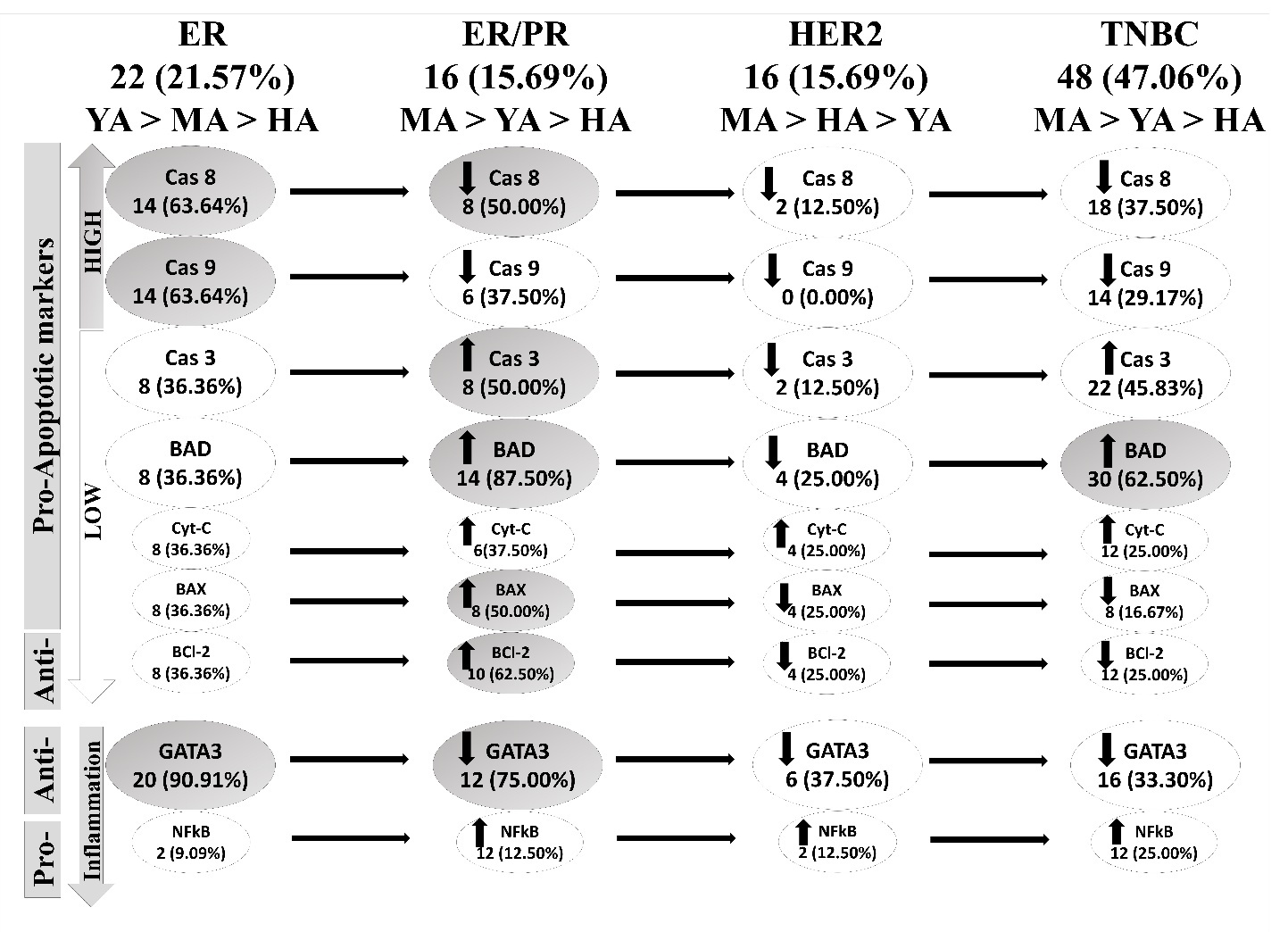


Supplementary Figure 6: Expression profile of Cas 8, Cas 9, Cas 3, BAD, Cyt-C, BAX, BCL-2, GATA3 and NFkB proteins across breast cancer subtypes of tumors of Nigerian breast cancer patients


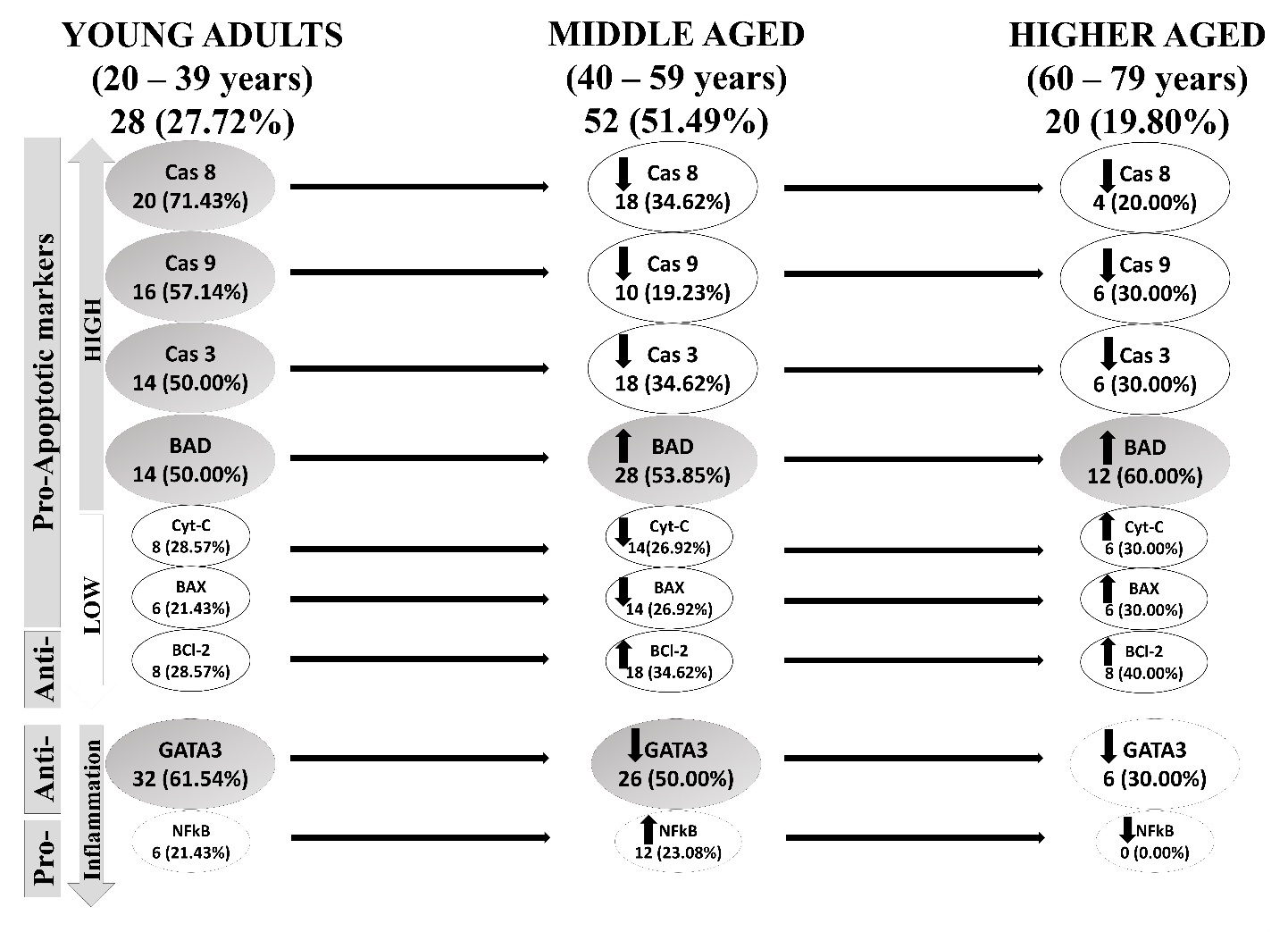


Supplementary Figure 7: Expression profile of Cas 8, Cas 9, Cas 3, BAD, Cyt-C, BAX, BCL-2, GATA3 and NFkB proteins across age groups of tumors of Nigerian breast cancer patients
